# ETV4 Mediated Tumor‐Associated Neutrophil Infiltration Facilitates Lymphangiogenesis and Lymphatic Metastasis of Bladder Cancer

**DOI:** 10.1002/advs.202205613

**Published:** 2023-01-20

**Authors:** Qiang Zhang, Sen Liu, Hongjin Wang, Kanghua Xiao, Junlin Lu, Siting Chen, Ming Huang, Ruihui Xie, Tianxin Lin, Xu Chen

**Affiliations:** ^1^ Department of Urology Sun Yat‐sen Memorial Hospital Sun Yat‐sen University Guangzhou Guangdong 510000 P. R. China; ^2^ Guangdong Provincial Key Laboratory of Malignant Tumor Epigenetics and Gene Regulation Sun Yat‐sen Memorial Hospital Sun Yat‐sen University Guangzhou Guangdong 510000 P. R. China; ^3^ Guangdong Provincial Clinical Research Center for Urological Diseases Guangzhou Guangdong 510000 P. R. China

**Keywords:** bladder cancer, lymphatic metastasis, tumor‐associated neutrophil

## Abstract

As a key step of tumor lymphatic metastasis, lymphangiogenesis is regulated by VEGFC‐VEGFR3 signaling pathway mediated by immune cells, mainly macrophages, in the tumor microenvironment. However, little is known whether tumor associated neutrophils are involved in lymphangiogenesis. Here, it is found that TANs infiltration is increased in LN‐metastatic BCa and is associated with poor prognosis. Neutrophil depletion results in significant reduction in popliteal LN metastasis and lymphangiogenesis. Mechanistically, transcription factor ETV4 enhances BCa cells‐derived CXCL1/8 to recruit TANs, leading to the increase of VEGFA and MMP9 from TANs, and then facilitating lymphangiogenesis and LN metastasis of BCa. Moreover, phosphorylation of ETV4 at tyrosine 392 by tyrosine kinase PTK6 increases nuclear translocation of ETV4 and is essential for its function in BCa. Overall, the findings reveal a novel mechanism of how tumor cells regulate TANs‐induced lymphangiogenesis and LN metastasis and identify ETV4 as a therapeutic target of LN metastasis in BCa.

## Graphical abstract

Tumor‐associated neutrophil (TANs) infiltration is increased in lymph node (LN)‐metastatic bladder cancer and is associated with poor prognosis. Neutrophil depletion results in significant reduction in popliteal LN metastasis and lymphangiogenesis. Mechanistically, protein tyrosine kinase 6 (PTK6)‐mediated phosphorylation of ETS variant transcription factor (ETV4) enhances bladder cancer (BCa) cells‐derived chemokine C‐X‐C motif ligand (CXCL)1/8 to recruit TANs, leading to the increase of vascular endothelial growth factor A (VEGFA) and matrix metalloproteinase‐9 (MMP9), and then facilitating lymphangiogenesis and LN metastasis of bladder cancer.

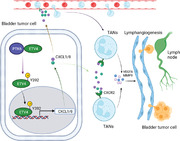

## 1 Background

Bladder cancer (BCa) is the most common cancer of urinary system worldwide, with approximately 573 000 new cases and 213 000 deaths in 2020.^[^ ^1^ ^]^ Lymph node (LN) metastasis is the main metastatic pattern of BCa and the leading cause of mortality, with an increased death rate of patients from 18.6% to 77.6% within 5 years.^[^ ^2^, ^3^ ^]^ Metastasis into the lymphatic circulation and invasion into the LNs of tumor cells are greatly facilitated by tumor lymphangiogenesis, a process that generates new lymphatic vessels.^[^ ^4^ ^]^ Lymphangiogenesis accelerates LN metastasis of various tumors by serving as channels for invaded tumor cells, promoting survival of cancer stem cells and suppressing antitumor immunity.^[^ ^5^ ^]^ Growth factors secreted by tumor cells, immune cells, and other components in the tumor microenvironment (TME), such as VEGFA, VEGFC, and VEGFD, induce lymphangiogenesis in primary tumors and draining LNs.^[^ ^6^, ^7^, ^8^, ^9^ ^]^

Various tumor‐associated immune cells play an important role in lymphangiogenesis. It is reported tumor‐associated macrophages, mast cells and basophils promote lymphangiogenesis by regulating VEGFC‐VEGFR3 signaling.^[^ ^10^, ^11^, ^12^, ^13^, ^14^ ^]^ Thus, investigating how tumor‐associated immune cells regulate lymphangiogenesis provides prognostic markers and therapeutic targets for LN metastasis of BCa.

Neutrophils have long been known to serve as the early responder to infectious agents in innate immunity and adaptive immune responses in peripheral blood.^[^ ^15^ ^]^ Not only do neutrophils response to acute inflammation but they are also an important component of TME.^[^ ^16^, ^17^, ^18^ ^]^ TANs express abundant chemokine receptors CXCR1 and CXCR2, which are important for their recruitment to TME, where chemokines (such as CXCL1, CXCL2, CXCL5, CXCL6, and CXCL8) are secreted by cancer cells and infiltrated immune cells.^[^ ^19^ ^]^ TANs may exhibit an anti‐tumor effect by releasing thrombospondin‐1 (TSP‐1), elastase, tumor necrosis factor (TNF), nitric oxide (NO) and reactive oxygen species (ROS), but more frequently exert a protumor effect by promoting immunosuppression, tumor cell proliferation, genetic instability, angiogenesis, and metastasis.^[^ ^20^, ^21^, ^22^, ^23^, ^24^, ^25^ ^]^ In addition to angiogenesis, accumulated neutrophils also contribute to lymphangiogenesis via regulating VEGFA bioactivity at sites of inflammation.^[^ ^26^ ^]^ However, little is known whether infiltrated neutrophils regulate lymphangiogenesis in TME.

Here, we report that TANs promote LN metastasis and predict poor prognosis of BCa. Depletion of neutrophils significantly inhibits lymphangiogenesis in footpad tumor and LN metastasis of BCa cells. In addition, transcription factor ETV4 increases CXCL1 and CCL8 secretion in BCa cells, resulting in increased MMP9 and VEGFA, but not VEGFC production in TANs. Those findings reveal a novel mechanism of how interaction between tumor cells and TANs in lymphangiogenesis and LN metastasis.

## 2 Results

### 2.1 TANs Correlate with LN Metastasis and Poor Prognosis in BCa

**Figure 1:**
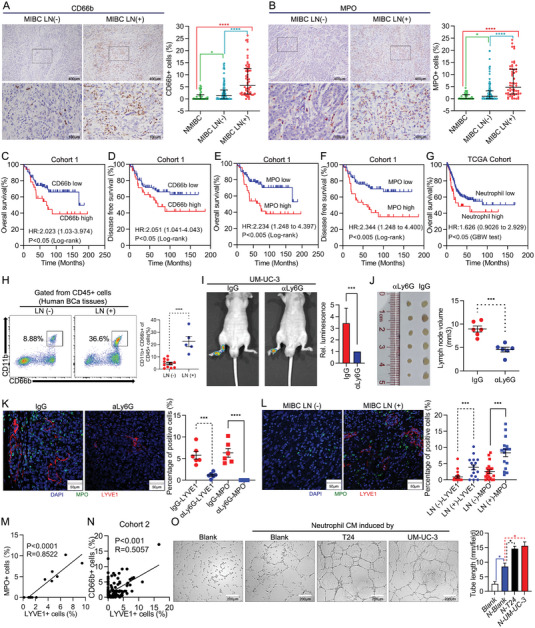
Tumor‐associated neutrophils promote lymphangiogenesis and LN metastasis in BCa. Representative IHC images straining for A) CD66b and B) MPO in MIBC LN (‐) and MIBC LN (+) BCa tissues (left). The quantification of A) CD66b^+^ and B) MPO ^+^ cells infiltrated in NMIBC (*n* = 26), MIBC LN (‐) (*n* = 84) and MIBC LN (+) (*n* = 50) BCa tissues (right). Kaplan‐Meier survival analysis of C) OS and D) DFS of BCa patients with high level (*n* = 76) versus low level (*n* = 84) infiltration of CD66b^+^cells in cohort 1. Kaplan‐Meier survival analysis of E) OS and F) DFS of BCa patients with high level (*n* = 71) versus low level (*n* = 89) infiltration of MPO^+^ cells in cohort 1. G) Kaplan‐Meier survival analysis of OS of BCa patients’ high level (*n* = 49) versus low level (*n* = 316) infiltration of TANs in TCGA cohort. H) Flow cytometry analysis of CD45^+^CD11b^+^CD66b^+^ neutrophils from fresh surgically excised BCa tissues (*n* = 16). I) Representative bioluminescence images (left) and histogram analysis (right) of the popliteal metastatic LNs from nude mice treated with IgG or neutralizing antibody of Ly6G (1A8, 300 µg per mouse). J) Images of the dissected popliteal LNs (left) and analysis (right) of the LN volume (*n* = 12). K,L) Representative immunofluorescence images (left) staining for MPO and LYVE1 and quantification (right) of MPO^+^ and LYVE1^+^ cells infiltrated in K) IgG‐ and aLy6G‐ treated footpad tumor or L) LN (‐) and LN (+) human BCa tissues. M) Correlation analysis between immunofluorescent detected MPO ^+^ and LYVE1^+^ cells in IgG‐ and aLy6G‐ treated footpad tumor (*n* = 12). N) Correlation analysis between CD66b^+^ cells and LYVE1^+^ cells in cohort 2 (*n* = 173). O) Representative images of tube formation assay of HLECs incubated with CM from neutrophils treated with the indicated supernatant for 6 h, histograms show means and SD for tube length. Statistical significance was assessed using two‐tailed t tests. *p < 0.05, ***p < 0.01, ***p < 0.001, ****p < 0.0001.

To investigate clinical significance of TANs infiltration in LN metastasis of BCa, we performed immunohistochemistry staining for CD66b or MPO‐specific markers for neutrophils‐in the tumor tissues of 207 patients with BCa. We found that higher level of CD66b^+^ or MPO^+^ neutrophil infiltration was positively correlated with high grade and muscle‐invasive BCa (MIBC) (Figure S1A,B and Tables S1 and S2, Supporting Information). Moreover, patients with LN‐metastatic MIBC presented significantly higher level of CD66b^+^ or MPO^+^ neutrophil infiltration than those with LN‐negative MIBC (**Figure** **1**A,B and Tables S1 and S2, Supporting Information). To further confirm the TANs infiltration in BCa tissue, CD16 was also stained. Consistent with previous findings, CD16^+^ cell infiltration positively correlated with MIBC and LN‐positive diseases (Figure S1C, Supporting Information). Additionally, gene‐set enrichment analysis (GSEA) suggested that neutrophil chemotaxis, extravasation and migration pathways were upregulated in LN‐metastatic BCa compared to LN‐negative BCa (Figure S1D, Supporting Information). Furthermore, higher level of CD66b^+^ or MPO^+^ neutrophil infiltration was associated with poorer overall survival (OS) and disease‐free survival (DFS) in BCa patients (Figure 1C–F). Moreover, univariate and multivariate Cox regression analyses confirmed that high CD66b^+^ or MPO^+^ neutrophil infiltration in BCa tissues was an independent prognostic factor for shorter OS and DFS (Tables S3–S6, Supporting Information).

Similarly, the analysis of The Cancer Genome Atlas (TCGA) database through CIBERSORTx (https://cibersortx.stanford.edu) also revealed that patients with higher level of neutrophil infiltration had reduced OS (Figure 1G). Additionally, the analysis of TCGA database, Lee Bladder Cohort and BLAVERI bladder Cohort from the Oncomine database indicated that higher levels of MPO expression was correlated with shortened OS (Figure S1E–G, Supporting Information); this was also corroborated for various types of cancer in TCGA database (Figure S1H–L, Supporting Information).

To further validate correlation between TANs infiltration and LN metastasis of BCa, we obtained fresh surgically excised bladder tumor tissues and proceeded flow cytometry analysis of neutrophils. Significantly increased CD11b^+^CD66b^+^ (gated from CD45^+^ cells) neutrophils were observed in tumor tissues of the patients with LN‐metastatic BCa compared to those with LN‐negative BCa (Figure 1H). Together, these results suggest that TANs infiltration is positive correlation with LN metastasis and predicts a poor prognosis of BCa.

### 2.2 TANs Promote LN Metastasis of BCa in Mice

To determine whether TANs infiltration increases with tumor progress, we inoculated human bladder cancer UM‐UC‐3/luc cells into the footpads of nude mice. As shown in Figure S2A (Supporting Information), TANs accumulated at footpad tumor over time, suggesting TANs might affect BCa progress in mice. To determine the function of TANs in LN metastasis of BCa, neutrophils were depleted using anti‐Ly6G antibody. Treatment was initiated before tumor cells were inoculated and continued until mice developed overt LN metastatic disease (Figure S2B, Supporting Information). Flow cytometry and IHC analysis revealed that neutrophils were efficiently depleted in the peripheral blood and footpad (Figure S2C,D, Supporting Information). We further performed Gr1 staining to confirm neutrophils in mouse tissues. Considering that Ly6C is also expressed in some monocytes,^[^ ^27^ ^]^ Gr1 positive cells were significantly fewer, but not disappeared, in *α*Ly6G group (Figure S2E, Supporting Information). Interestingly, neutrophil depletion resulted in a significant reduction in popliteal LN metastasis, as determined by the in vivo imaging system (IVIS) (Figure 1I). Moreover, smaller volume of popliteal LN (Mean volume 4.555 mm^3^ vs 8.957 mm^3^) and decreased LN metastasis rate (16.7% vs 66.6%) were observed in a neutrophils‐depleted group compared with control group (Figure 1J and Figure S2F, Supporting Information). Overall, we discover that TANs promote LN metastasis of BCa in vivo.

### 2.3 Neutrophil Contributes to Lymphangiogenesis in BCa

Since neutrophils recruited to sites of inflammation coordinate lymphangiogenesis,^[^ ^26^ ^]^ we hypothesized that infiltrated TANs might contribute to lymphangiogenesis in tumor. To validate this hypothesis, we performed IHC analysis of LYVE1‐a lymphatic endothelial cell‐specific marker. Neutrophil depletion obviously decreased lymphangiogenesis in footpad tumor (Figure 1K and Figure S2G, Supporting Information). Moreover, a positive correlation was observed between the infiltration neutrophils to the aggregation of lymphatic endothelial cells around them (Figure 1K,M). In addition, IF staining of MPO and LYVE1 revealed that MPO^+^ neutrophils and microlymphatic vessel density (MLD) were all stronger in LN‐metastatic BCa than in LN‐negative BCa (Figure 1L). Furthermore, we observed significant positive correlation between neutrophil infiltration and MLD in human BCa tissues (Figure 1N). To further investigate whether TANs directly regulate lymphangiogenesis, we isolated neutrophils from peripheral blood of patients with BCa (Figure S2G, Supporting Information) and proceeded tube formation assay of HLECs in vitro. Interestingly, neutrophil culture medium (CM) slightly increased tube formation of HLECs, while T24 or UM‐UC‐3 cells‐stimulated‐neutrophil CM significantly enhanced this effect (Figure 1O). Taken together, those results demonstrate that infiltrated neutrophils promote lymphangiogenesis and LN metastasis of BCa.

### 2.4 TANs‐Derived VEGFA and MMP9 Are Essential for Lymphangiogenesis

**Figure 2:**
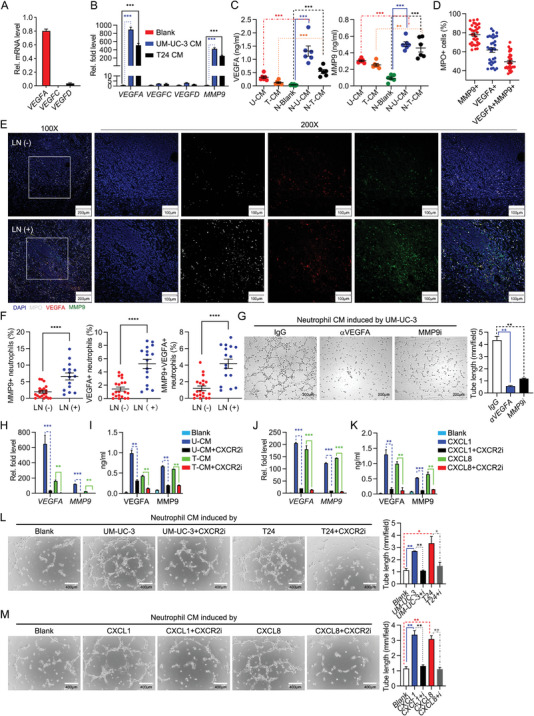
BCa cell‐derived CXCL1/8 promote TANs‐modulated lymphangiogenesis. A) qRT‐PCR analysis of *VEGFA*, *VEGFC* and *VEGFD* mRNA in neutrophils. B) qRT‐PCR analysis of *VEGFA*, *VEGFC*, *VEGFD* and *MMP9* mRNA in neutrophils treated with the culture media (CM) from UM‐UC‐3 and T24 for 3 h, respectively. C) ELISA analysis of VEGFA and MMP9 in the supernatants of UM‐UC‐3, T24 and neutrophils treated with the indicated supernatant for 12 h, respectively. D) Percentage of MMP9^+^, VEGFA^+^ and MMP9^+^ VEGFA^+^ cells in MPO^+^ cells. E) Representative immunofluorescence images staining for MPO, VEGFA and MMP9 in LN (‐) (*n* = 20) and LN (+) (*n* = 16) human BCa tissues. F) Quantification of MPO^+^MMP9^+^, MPO^+^VEGFA^+^ and MPO^+^ MMP9^+^ VEGFA^+^ cells in LN (‐) and LN (+) human BCa tissues. G) Representative images of tube formation assay of HLECs incubated with CM from UM‐UC‐3‐induced neutrophils treated with IgG, neutralizing antibody of VEGFA (100 ng mL^−1^) or inhibitor of MMP9 (MMP‐9‐IN‐1, 20 × 10^−6^ m) for 6 h, histograms show means and SD for tube length. H) qRT‐PCR analysis of VEGFA and MMP9 mRNA in neutrophils incubated with UM‐UC‐3 or T24 CM and treated with inhibitor of CXCR2 (SB225002, 50 × 10^−6^ m) for 3 h, respectively. I) ELISA analysis of VEGFA and MMP9 in the supernatants of neutrophils incubated with above treatment for 12 h. J) qRT‐PCR analysis of VEGFA and MMP9 mRNA in neutrophils incubated with CXCL1 or CXCL8 (10 ng mL^−1^) and treated with inhibitor of CXCR2 (SB225002, 50 × 10^−6^ m) for 3 h, respectively. K) ELISA analysis of VEGFA and MMP9 in the supernatants of neutrophils incubated with above treatment for 12 h. L,M) Representative images of tube formation assay of HLECs incubated with CM from neutrophils with the indicated treatment for 6 h, histograms show means and SD for tube length. Statistical significance was assessed using two‐tailed t tests or one‐way ANOVA. **p < 0.01, ***p < 0.001, ****p < 0.0001. Data are representative of two (C,I,K,L, M) or three independent experiments (mean ± S.D. in A,B,H,J).

To understand the mechanism how infiltrated TANs facilitate lymphangiogenesis, we examined VEGFs expression in isolated neutrophils. Unexpectedly, quantitative real‐time PCR (qRT‐PCR) releveled that detectable *VEGFA*, while little *VEGFC* and *VEGFD* were measured in freshly isolated neutrophils (**Figure** **2**A). In addition, expression of *VEGFA* and *MMP9* in neutrophils was significantly increased, but not *VEGFC* or *VEGFD* after stimulating by CM of T24 or UM‐UC‐3 cells (Figure 2B). Enzyme‐linked immunosorbent assay (ELISA) analysis revealed that production of VEGFA and MMP9 in untreated neutrophils was much lower than in BCa cells (Figure 2C). However, production of VEGFA and MMP9 in neutrophils was significantly increased to 2‐3 folds of BCa cells after stimulating by CM of T24 or UM‐UC‐3 cells (Figure 2C). Furthermore, IF staining of MPO, MMP9 and VEGFA revealed that main of the MMP9 and almost half of the VEGFA were produced by TANs (Figure 2D–F). Previous reports identified that neutrophils contributed to inflammatory lymphangiogenesis by increasing VEGFA bioavailability and bioactivity via the secretion of MMP9.^[^ ^26^ ^]^ Consistently, blocking VEGFA by its neutralizing antibody or inhibiting MMP9 activity by its inhibitor effectively suppressed UM‐UC‐3 cells‐stimulated‐neutrophil‐mediated tube formation of HLECs (Figure 2G), suggesting neutrophil‐derived VEGFA and MMP9 are essential for tube formation of HLECs.

### 2.5 CXCL1/8‐CXCR2 Axis Is Crucial for Neutrophil‐Mediated Lymphangiogenesis

It is reported that CXCL1/8‐CXCR2 and ERK/JNK pathway are relevant with neutrophil‐derived VEGFA,^[^ ^28^, ^29^ ^]^ while CXCL1 and CXCL8 are abundant in BCa,^[^ ^30^, ^31^, ^32^, ^33^ ^]^ we hypothesized that BCa cells‐derived CXCL1 or CXCL8 might cooperate with CXCR2 of neutrophil to activate ERK/JNK pathway, leading to increase the production of VEGFA and MMP9. Expression of *VEGFA* or *MMP*9 and production of VEGFA or MMP9 were measured after challenged with CM of UM‐UC‐3 cells, with or without inhibitors of ERK and JNK. ERK or JNK inhibition significantly counteracted the increase in expression of VEGF‐A and MMP9 induced by CM of UM‐UC‐3 cells, with comparable efficacy (Figure S3A,B, Supporting Information). In addition, phosphorylation of ERK and JNK was induced by CM of T24 or UM‐UC‐3 cells (Figure S3C, Supporting Information). Moreover, CXCR2 inhibitor (SB225002) markedly impaired T24 or UM‐UC‐3 cells‐induced expression of *VEGFA* or *MMP9* and production of VEGFA or MMP9 (Figure 2H,I). Consistently, both CXCL1 and CXCL8 significantly induced expression of VEGFA and MMP9, while inhibition of CXCR2 blocked this effect (Figure 2J,K). Consistent with the results from gene induction analysis, phosphorylation of ERK and JNK induced by BCa cells CM or CXCL1/8 were decreased by an inhibitor of CXCR2 (Figure S3C,D, Supporting Information). Moreover, inhibition of CXCR2 also impaired BCa cells or CXCL1/8‐stimulated‐neutrophils induced tube formation of HLECs (Figure 2L,M).

Regulation of CXCR2 expression in neutrophils may affect the protumorigenic functions under diverse conditions. At cellular level, we detected CXCR2 protein expression in neutrophil after stimulating by conditioned medium of T24 or UM‐UC‐3 cells and CXCL1/8. After stimulating by CM or CXCL1/8, CXCR2 expression was not changed (Figure S3C,D, Supporting Information). At tissue level, IF staining of CXCR2 and MPO is performed. More MPO^+^ CXCR2^+^ neutrophils were identified in tumor tissue than in normal adjacent tissue. While much more MPO^+^ CXCR2^+^ neutrophils are found in LN(+) tumor tissue than in LN(‐) tumor tissue (Figure S3E, Supporting Information). However, there is no difference in the normal adjacent tissue between the LN(+) and LN(‐) patients (Figure S3E, Supporting Information). Based on these results, CXCR2 expression of neutrophil is upregulated in tumor microenvironment, especially in LN+ tumor. These data collectively indicate that CXCL1/8‐CXCR2 axis is crucial for neutrophil‐mediated lymphangiogenesis by promoting VEGFA and MMP9 expression. These data collectively indicate that CXCL1/8‐CXCR2 axis is crucial for neutrophil‐mediated lymphangiogenesis by promoting VEGFA and MMP9 expression.

### 2.6 ETV4 Regulates CXCL1/8 Transcription in BCa Cells

**Figure 3:**
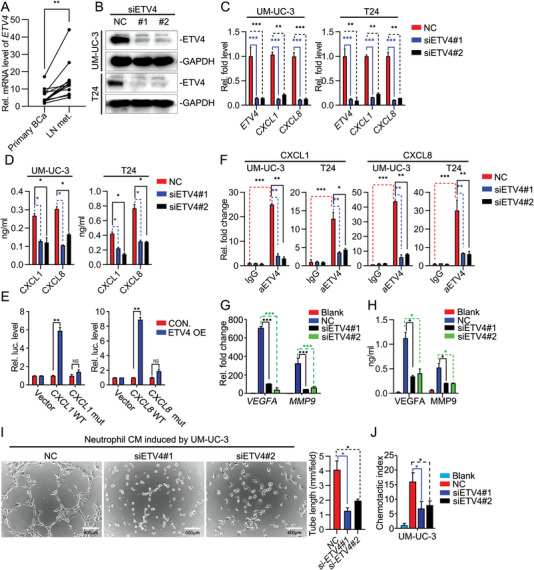
ETV4 is a transcriptional regulator of CXCL1/8 in BCa cells. A) qRT‐PCR analysis of *ETV4* mRNA in paired primary tumors and LN metastases from BCa patients (*n* = 12). B) Immunoblot analysis of ETV4 and GAPDH in UM‐UC‐3 and T24 cells transfected with siRNA for 48 h. C) qRT‐PCR analysis of *ETV4*, *CXCL1* and *CXCL8* mRNA in UM‐UC‐3 and T24 cells transfected with siRNA for 48 h. D) ELISA analysis of CXCL1 and CXCL8 in the supernatants of UM‐UC‐3 and T24 cells transfected with siRNA for 48 h. E) Luciferase reporter assays analyzing the activity of wildtype (WT) and mutation (mut) CXCL1 and CXCL8 promoter. F) ChIP analysis of *CXCL1* and *CXCL8* genes in UM‐UC‐3 and T24 infected with siRNA for 48 h. G) qRT‐PCR analysis of *VEGFA* and *MMP9* mRNA in neutrophils incubated with indicated UM‐UC‐3 CM. H) ELISA analysis of VEGFA and MMP9 in the supernatants of neutrophils treated with the indicated supernatant for 12 h. I) Representative images of tube formation assay of HLECs incubated with CM from neutrophils with the indicated treatment for 6 h, histograms show means and SD for tube length. J) Chemotactic index analysis of neutrophils attracted by indicated UM‐UC‐3 CM. Statistical significance was assessed using two‐tailed t tests or one‐way ANOVA. *p <0.05, **p < 0.01, ***p < 0.001. Data are representative of two (B, D, E, F, H,I) or three independent experiments (mean ± S.D. in C,G).

Since BCa cell‐derived CXCL1 and CXCL8 are essential for neutrophil‐modulated lymphangiogenesis, we hypothesized that their regulator gene might also play an important role. We analyzed promoter and enhancer of CXCL1 and CXCL8 using GeneHancer, a regulatory element database within the GeneCards Suite (https://www.genecards.org/). We found 14 and 33 transcription factors might bind to promoter or enhancer of CXCL1 and CXCL8, respectively. By analyzing RNA‐sequencing date from TCGA database, we identified that only ETV4, a potential CXCL8 regulator, was highly expressed in tumor tissues compared to normal adjacent tissues (NATs) (Figure S4A,B, Supporting Information). Similarly, the analysis of Lee Bladder Cohort from the Oncomine database also indicated that ETV4 was upregulated in tumor tissues compared to normal adjacent tissues (Figure S4C, Supporting Information). RT‐qPCR and western blot analysis confirmed ETV4 overexpression in BCa tissues from patients compared with the corresponding NATs (Figure S4D,E, Supporting Information). Analysis of the paired primary tumors and metastatic LNs from the same patients further confirmed the obvious elevation of ETV4 in metastatic LNs (**Figure** **3**A). Next, we investigated whether ETV4 was a CXCL8 regulator. We designed two siRNAs of ETV4, both of which potently downregulated the mRNA and protein levels of ETV4 in UM‐UC‐3 or T24 cells (Figure 3B,C). Knockdown of ETV4 by these two siRNAs significantly inhibited expression of *CXCL8* in UM‐UC‐3 or T24 cells (Figure 3B). Unexpectedly, knockdown of ETV4 also impaired expression of *CXCL1*, rather than other neutrophil recruitment‐associated chemokines or cytokines, including *CXCL2*, *CXCL5*, *CXCL6*, *IL1B*, *IL17A* and *TNFA* (Figure S4F, Supporting Information). In addition, the knockdown of ETV4 decreased CXCL1 and CXCL8 secretion, as determined by ELISA (Figure 3D). To further confirm that ETV4 drives transcription of *CXCL1* and *CXCL8*, we cloned 200 bp around ETV4 binding sites of *CXCL1* and *CXCL8* into the pGL3‐Basic luciferase vector, and made constructs with mutations in the ETV4 binding sites, and performed luciferase reporter assays. Interestingly, ETV4 potently activated the luciferase activity of *CXCL1* and *CXCL8* promoter, which was substantially impaired by mutation of ETV4 binding site (Figure 3E). Results from the chromatin immunoprecipitation (ChIP) assay demonstrated that ETV4 directly bound to promoter of CXCL1 and CXCL8, while knockdown of ETV4 decreased the enrichment (Figure 3F and Figure S4G, Supporting Information). These results suggest that ETV4 directly binds to promoter of *CXCL1* and *CXCL8* and drives transcriptional activation of *CXCL1* and *CXCL8* gene.

### 2.7 Knockdown of ETV4 Inhibits TANs Recruitment and TANs‐Mediated Tube Formation of HLECs In Vitro

**Figure 4:**
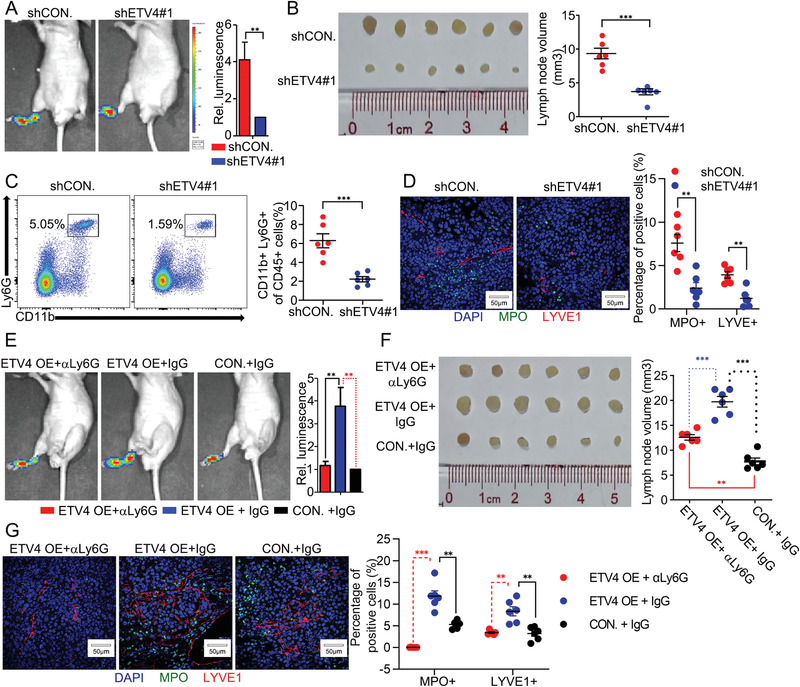
ETV4 enhances LN metastasis of BCa in vivo via increasing TANs recruitment. A) Representative bioluminescence images (left) and histogram analysis (right) of the popliteal metastatic LNs from nude mice injected with control shRNA (shCON.) or shETV4#1 stably transfected UM‐UC‐3 cells for 5 weeks. The relative luminescence is 1 in shETV4#1. B) Images of the dissected popliteal LNs (left) from indicated nude mice and analysis (right) of the LN volume. C) Flow cytometry analysis of CD45^+^CD11b^+^Ly6G^+^ neutrophils from fresh surgically excised footpad tumor tissues. D) Representative immunofluorescence images (left) staining for MPO and LYVE1 and quantification (right) of MPO^+^ and LYVE1^+^ cells infiltrated in footpad tumor from indicated nude mice. E) Representative bioluminescence images (left) and histogram analysis (right) of the popliteal metastatic LNs from nude mice injected with the empty vector (CON.) or ETV4 overexpression vector stably transfected UM‐UC‐3 cells and IgG or neutralizing antibody of Ly6G for 4 weeks. F) Images of the dissected popliteal LNs (left) from indicated nude mice and analysis (right) of the LN volume. G) Representative immunofluorescence images (left) staining for MPO and LYVE1 and quantification (right) of MPO^+^ and LYVE1 ^+^ cells infiltrated in footpad tumor from indicated nude mice. Statistical significance was assessed using two‐tailed t tests or one‐way ANOVA. *p < 0.05, **p < 0.01, ***p < 0.001.

Next, we explored the role of ETV4 in BCa cells on TANs. Knockdown of ETV4 decreased UM‐UC‐3 or T24 cells‐induced expression of VEGFA and MMP9 in TANs (Figure 3G,H and Figure S5A,B, Supporting Information). Consistently, knockdown of ETV4 impaired UM‐UC‐3 cells‐stimulated‐TANs induced tube formation of HLECs (Figure 3I). Since the secretion of CXCL1 and CXCL8 by cancer cells promoted TANs recruitment to the tumor sites, we hypothesized that ETV4 might promote TANs accumulation. TANs were added to the upper chamber, while the supernatant of UM‐UC‐3 cells was added to the lower chamber. As expected, the migratory response of TANs to the supernatant of ETV4‐knockdown cells was suppressed (Figure 4J). Collectively, these data reveal that knockdown of ETV4 in BCa cells decrease TANs recruitment and TANs‐mediated tube formation of HLECs in vitro.

### 2.8 ETV4 Enhances LN Metastasis of BCa In Vivo via Increasing TANs Recruitment

To further investigate the role of ETV4 in regulating LN metastasis in vivo, the UM‐UC‐3/luc cells with the indicated lentiviral transfection were inoculated into the footpads of nude mice. Knockdown of ETV4 resulted in a significant reduction in popliteal LN metastasis and shortened animal survival (**Figure** **4**A and Figure S6A, Supporting Information). Consistently, decreased volume of popliteal LN and reduced LN metastasis rate were observed in ETV4‐knockdown group (Figure 4B and Figure S6B, Supporting Information). These findings demonstrated knockdown of ETV4 alleviated the LN metastasis of BCa cells. Moreover, flow cytometry analyses showed that ETV4 knockdown decreased the percentages of CD11b^+^Ly6G^+^ TANs, but not CD11b^+^F4/80^+^ TAMs in the footpad tumor (Figure 4C and Figure S6C, Supporting Information). The changes of neutrophils in footpad tumor were not caused by altered biogenesis, since their abundance in the spleen of the mice was negligibly changed (Figure S6D, Supporting Information). Noticeably, knockdown of ETV4 significantly decreased lymphangiogenesis in footpad tumor (Figure 4D). To further clarify these, we constructed the stably ETV4‐overexpressing UM‐UC‐3 cell line by lentiviral transfection. We found that overexpression of ETV4 wild type (WT) enhanced LN metastasis of UM‐UC‐3 cells, TANs recruitment and lymphangiogenesis in vivo. However, when TANs were specifically depleted in the mice by the anti‐Ly6G antibody before BCa cells injection, the pro‐LN metastasis effect of ETV4 was significantly attenuated, including the LN metastasis rate and the volume of popliteal LNs (Figure 4E,F and Figure S6E, Supporting Information). In addition, overexpression of ETV4 enhanced lymphangiogenesis in footpad of mice, which was mostly inhibited by depleting TANs (Figure 4G). It suggests that the promoting function of ETV4 in lymphangiogenesis is also mediated by increased TANs recruitment.

### 2.9 ETV4 Promotes Migration and Invasion of BCa Cells In Vitro

Interestingly, the volume of popliteal LN (mean volume 12.58 mm^3^) and LN metastasis rate (66.67%) in (ETV4 OE + aLy6G) group were still larger and higher than the volume of popliteal LN (mean volume 7.76 mm^3^) and LN metastasis rate (50%) in (CON. + IgG) group (Figure 4F and Figure S6E, Supporting Information), suggesting the pro‐LN‐metastatic effect of ETV4 was not only depending on TANs. To further investigate the pro‐LN‐metastatic effect of ETV4, we proceeded cell metastasis assay in vitro. We observed that ETV4 knockdown inhibited, while ETV4 overexpression enhanced migration and invasion abilities of BCa cells (Figures S7A,B and S8E,F, Supporting Information). Meanwhile, annexin V/PI apoptotic assay revealed that ETV4 knockdown did not affect the apoptosis of BCa cells (Figure S7C,D, Supporting Information). Western blotting, and immunofluorescence analyses revealed that E‐cadherin was upregulated, whereas the expression levels of N‐cadherin and SNAIL were downregulated in ETV4‐knockdown cells (Figure S7E,F, Supporting Information). Similarly, immunoblotting analyses revealed that ETV4 overexpression upregulated the expression levels of N‐cadherin and SNAIL, whereas downregulated the expression of E‐cadherin (Figure S8G, Supporting Information). Collectively, those results suggest that ETV4 promotes migration and invasion of BCa cells in vitro.

### 2.10 Y392 of ETV4 Is Required for the Pro‐LN‐Metastatic Effect

**Figure 5:**
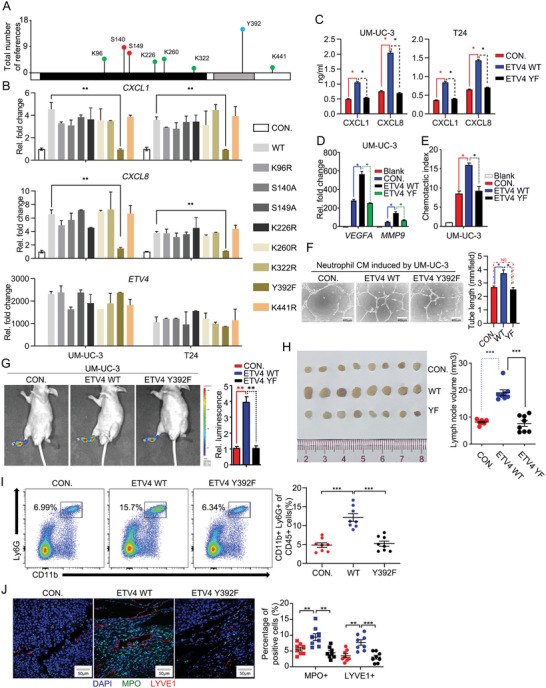
Y392 of ETV4 is required for the pro‐LN‐metastatic effect. A) Quantification of published post‐translational modification sites of ETV4. B) qRT‐PCR analysis of *ETV4*, *CXLC1*, *CXCL8* mRNA in ETV4 WT‐ or MUT‐overexpressed UM‐UC‐3 cells. C) ELISA analysis of CXCL1 and CXCL8 in the supernatants of ETV4 WT‐ or Y392F‐overexpressed UM‐UC‐3 and T24 cells. D) qRT‐PCR analysis of *VEGFA* and *MMP9* mRNA in neutrophils incubated with indicated CM for 3 h. E) Chemotactic index analysis of neutrophils attracted by indicated UM‐UC‐3 CM. F) Representative images of tube formation assay of HLECs incubated with CM from neutrophils treated with the indicated supernatant for 6 h, histograms show means and SD for tube length. G) Representative bioluminescence images (left) and histogram analysis (right) of the popliteal metastatic LNs from the indicated nude mice. The relative luminescence is 1 in empty vector (CON.). H) Images of the dissected popliteal LNs (left) from nude mice injected with the CON and ETV4 WT or Y392F overexpression vector stably transfected UM‐UC‐3 cells for 4 weeks. Analysis (right) of the LN volume. I) Flow cytometry analysis of CD45^+^CD11b^+^Ly6G^+^ neutrophils from fresh surgically excised footpad tumor tissues. J) Representative immunofluorescence images (left) staining for MPO and LYVE1 and quantification (right) of MPO^+^ and LYVE1 ^+^ cells infiltrated in footpad tumor tissues from indicated nude mice. Statistical significance was assessed using two‐tailed t tests or one‐way ANOVA. *p < 0.05, **p < 0.01, ***p < 0.001. Data are representative of two (C, E,F) or three independent experiments (mean ± S.D. in B,D).

Next, we interrogated the mechanisms underlying ETV4‐regualted CXCL1/8 transcription. Since post‐translational modifications are critical to the function of proteins, we analyzed the types of modifications that might occur in ETV4 through PhosphoSitePlus (https://www.phosphosite.org/). ETV4 might be phosphorylated at S140, S149 and Y392, and ubiquitinated at K96, K226, K260, K322 and K441 (**Figure** **5**A). To explore the potential effect of those sites on ETV4, we made constructs with mutations at those sites, respectively. RT‐qPCR analyses showed that overexpression of ETV4 WT, K96R, K226R, K260R, K322R, K441R, S140A and S149A all improved expression of CXCL1 and CXCL8, but not Y392F (Figure 5B), suggesting Y392 was critical for ETV4. Analysis of primary sequences of *Homo sapiens* ETV4 and its homologues in 11 species suggested that Y392 of ETV4 was evolutionarily conserved (Figure S8A, Supporting Information). Interestingly, overexpression of ETV4 WT but not Y392F in BCa cells increased CXCL1 and CXCL8 secretion of BCa cells, expression of VEGFA and MMP9 in neutrophils, neutrophil‐induced tube formation of HLECs, and chemotaxis of neutrophils in vitro (Figure 5C–F). Furthermore, in vivo assays showed that overexpression of Y392F mutated ETV4 could not promote LN metastasis of UM‐UC‐3 cells, TANs recruitment and lymphangiogenesis compared with ETV4 WT, leading to the prolonged animal survival (Figure 5G–J and Figure S8B–D, Supporting Information). Besides, overexpression of ETV4 WT but not Y392F enhanced the migration and invasion abilities of BCa cells (Figure S8E,F, Supporting Information). Consistently, E‐cadherin was downregulated, whereas the expression levels of N‐cadherin and SNAIL were upregulated in ETV4‐overexpressed cells, but this effect was disappeared in Y392F mutated ETV4 cells (Figure S8G, Supporting Information). Taken together, these data demonstrate that Y392 is critical for ETV4 to promote LN metastasis of BCa cells.

### 2.11 Phosphorylation of ETV4 at Y392 by PTK6 Regulates the Nuclear Translocation of ETV4

**Figure 6:**
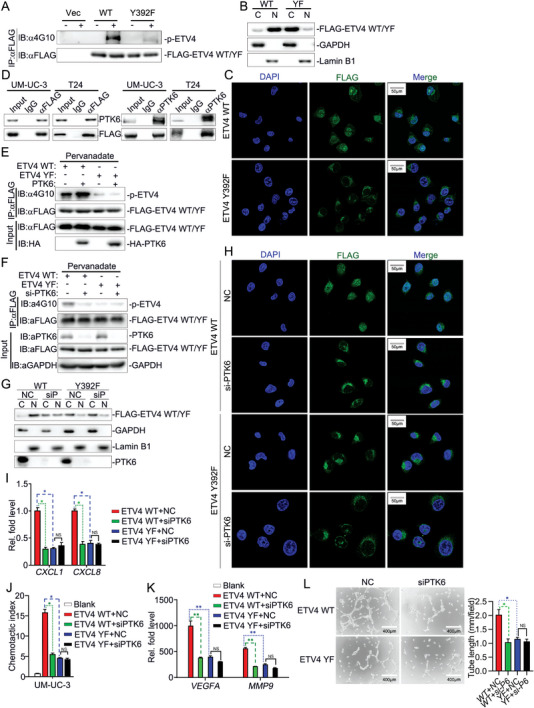
Phosphorylation of ETV4 at Y392 by PTK6 regulates the nuclear translocation of ETV4. A) Immunoprecipitation (with anti‐FLAG) and immunoblot analysis (with anti‐4G10 or anti‐FLAG) of the empty vector (CON.) and ETV4 WT or Y392F overexpression vector stably transfected UM‐UC‐3 cells in the absence or presence of pervanadate for 30 min. B) Immunoblot analysis of cytoplasmic (C) and nuclear (N) FLAG‐ETV4, GAPDH and Lamin B1 in ETV4 WT or Y392F overexpression vector stably transfected UM‐UC‐3 cells. C) Representative immunofluorescence images staining for FLAG‐ETV4 in ETV4 WT or Y392F overexpression vector stably transfected UM‐UC‐3 cells. D) Immunoprecipitation (with anti‐FLAG, anti‐PTK6 or control IgG) and immunoblot analysis (with anti‐FLAG or anti‐PTK6) of the empty vector (CON.) and ETV4 WT or Y392F overexpression vector stably transfected UM‐UC‐3 cells. E) Immunoprecipitation (with anti‐FLAG) and immunoblot analysis (with anti‐4G10, anti‐HA or anti‐FLAG) of HEK293 cells transfected with plasmids encoding HA‐PTK6 and FLAG‐ETV4 in the absence or presence of pervanadate for 30 min. F) Immunoprecipitation (with anti‐FLAG) and immunoblot analysis (with anti‐4G10, anti‐HA or anti‐FLAG) of UM‐UC‐3 cells that stably express FLAG‐ETV4 WT or Y392F, treated with NC or siPTK6 for 48 h. G) Immunoblot analysis of cytoplasmic (C) and nuclear (N) FLAG‐ETV4, PTK6, GAPDH and Lamin B1 in UM‐UC‐3 cells that stably express FLAG‐ETV4 WT or Y392F, treated with NC or siPTK6 for 48 h. H) Representative immunofluorescence images staining for FLAG‐ETV4 in UM‐UC‐3 cells that stably express FLAG‐ETV4 WT or Y392F, treated with NC or siPTK6 for 48 h. I) qRT‐PCR analysis of *CXCL1* and *CXCL8* mRNA in ETV4 WT or Y392F overexpression vector stably transfected UM‐UC‐3 cells, which were treated with NC or siPTK6 for 48 h. J) Chemotactic index analysis of neutrophils attracted by indicated UM‐UC‐3 CM. K) qRT‐PCR analysis of *VEGFA* and *MMP9* mRNA in neutrophils incubated with indicated UM‐UC‐3 CM. L) Representative images of tube formation assay of HLECs incubated with CM from neutrophils with the indicated treatment for 6 h, histograms show means and SD for tube length. Statistical significance was assessed using two‐tailed t tests or one‐way ANOVA. ^NS^ *p* >0.05, *p <0.05, **p < 0.01. Data are representative of two (A–G, J,L) or three independent experiments (mean ± S.D. in I,K).

Then, we investigated whether ETV4 was phosphorylated at Y392. Interestingly, ETV4 Y392F showed a markedly reduced level of tyrosine phosphorylation compared with ETV4 WT (**Figure** **6**A). Moreover, nuclear translocation of ETV4 Y392F was markedly decreased (Figure 6B,C), which suggested that ETV4 phosphorylation at Y392 had a possible role in regulating the nuclear translocation of ETV4. We set out to identify the tyrosine kinase that is responsible for ETV4 phosphorylation. Cytoplasmic proteins were separated to perform co‐Immunoprecipitation (Co‐IP) assays and mass spectrometry (MS). Tyrosine kinase PTK6 was identified as a potential ETV4‐interacting protein (Figure S9A, Supporting Information). Co‐IP assays suggested that ETV4 constitutively interacted with PTK6 (Figure 6D). Moreover, PTK6 overexpression promoted, while PTK6 knockdown reduced the phosphorylation of ETV4 in UM‐UC‐3 cells (Figure 6E,F). Furthermore, knockdown or inhibition of PTK6 did not significantly change nuclear translocation of ETV4 Y392F but did suppress nuclear translocation of ETV4 WT (Figure 6G,H and Figure S9B,C, Supporting Information). Consistently, PTK6 knockdown or inhibition of PTK6 by Tilfrinib in UM‐UC‐3 cells did not reduce ETV4 Y392 but did reduce ETV4 WT‐induced expression of *CXCL1* or *CXCL8* in UM‐UC‐3 cells and expression of *VEGFA* or *MMP9* in TANs (Figure 6I,K and Figure S9D,E, Supporting Information). In addition, PTK6 knockdown in UM‐UC‐3 cells did not affect ETV4 Y392F but did inhibit ETV4 WT‐associated tube formation of HLECs and chemotaxis of TANs in vitro (Figure 6J,L). Collectively, those results suggest that PTK6‐mediated ETV4 phosphorylation at Y392 promotes nuclear translocation of ETV4, which leads to promoted LN metastasis‐related function of ETV4.

### 2.12 ETV4 Expression Is Associated with Neutrophil Infiltration and Poor Prognosis in BCa

**Figure 7:**
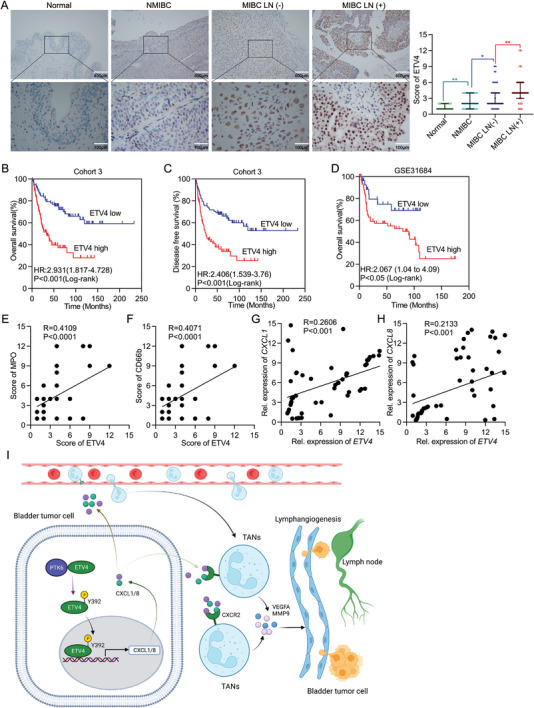
ETV4 expression is associated with neutrophil infiltration and poor prognosis in BCa. A) Representative IHC images straining for ETV4 (left) and quantification of ETV4 expression in NAT and indicated BCa tissues (right). B,C) Kaplan‐Meier survival analysis of B) OS and C) DFS of BCa patients with high level (*n* = 83) versus low level (*n* = 78) expression in cohort 3. D) Kaplan‐Meier survival analysis of OS of BCa patients with high level (*n* = 64) versus low level (*n* = 29) expression in GES31684 cohort from GEO database. E,F) Correlation analysis between ETV4 expression and CD66b^+^ or MPO^+^ cells in cohort 3 (*n* = 161). G,H) Correlation analysis between *ETV4* and *CXCL1* or *CXCL8* relative mRNA expression in cohort 4 (*n* = 62). I) Schematic diagram of ETV4‐mediated regulation of lymphangiogenesis and LN metastasis in BCa. Statistical significance was assessed using two‐tailed t tests or one‐way ANOVA.

We then assessed the clinical significance of ETV4 in LN metastasis of BCa. As shown by IHC, the protein expression levels of ETV4 were significantly upregulated in the LN‐metastatic BCa tissues, slightly elevated in LN‐negative BCa tissues, compared with normal adjacent tissues (**Figure** **7**A, Supporting Information). Additionally, ETV4 was positively correlated with muscle‐invasive and high‐grade BCa (Table S7, Supporting Information). Moreover, Kaplan‐Meier analysis demonstrated that high ETV4 expression was significantly associated with decreased OS and DFS (OS: cohort 3, HR = 2.931, 95% CI = 1.817 to 4.728, *p* < 0.001; DFS: cohort 3, HR = 2.406, 95% CI = 1.539 to 3.76, *p* < 0.001.) (Figure 7B,C). Importantly, univariate and multivariate Cox proportional hazards analyses showed that ETV4 expression was an independent prognostic factor for OS and DFS of BCa patients (Tables S8 and S9, Supporting Information). Similarly, the analysis of GSE31680 Cohort from GEO database indicated that higher levels of ETV4 expression were correlated with shortened OS (Figure 7D). Furthermore, our pathology results showed that ETV4 expression levels positively correlated with infiltrated MPO^+^ or CD66b^+^ TANs levels in the tumor tissues (Figure 7E,F). Consistently, we further observed *ETV4* mRNA expression levels positively correlated with *CXCL1* or *CXCL8* mRNA expression levels in the tumor tissues (Figure 7G,H). These results further verify that expression of ETV4 is positively correlated with CXCL1/8 expression and TANs infiltration, which predicts high rates of LN metastasis and poor prognosis outcome of BCa.

## 3 Discussion

In this study, we report that a role of neutrophils to promote LN metastasis of BCa cells by regulating lymphangiogenesis. Tumor cells‐secreted CXCL1 and CXCL8 recruit neutrophils and promote activation of ERK and JNK signaling pathways and expression of VEGFA and MMP9 in neutrophils, which induce tumor lymphangiogenesis and facilitate LN metastasis of tumor cells. Meanwhile, phosphorylation of ETV4 at Y392 by PTK6 promotes expression of CXCL1 and CXCL8, which enhances neutrophil recruitment, neutrophils‐mediated lymphangiogenesis, and LN metastasis of BCa cells.

The association of TANs and metastasis has recently received intensive attention. Dysregulation of TME frequently causes systemic inflammation, which is called cancer‐cell‐intrinsic inflammation.^[^ ^34^ ^]^ Abundant CXC chemokine (such as CXCL1, CXCL2 and CXCL8), growth factors (G‐CSF and GM‐CSF) and inflammatory cytokines (for example, IL‐6, IL‐1*β*, and IL‐17) produced by tumor cells, tumor‐associated stromal cells and tumor infiltrating leukocytes facilitate neutrophil infiltration.^[^ ^19^ ^]^ TANs participate in multiple metastatic cascades. TANs‐derived CXCL8 and IL17A trigger EMT by increasing expression of SNAIL, TWIST, and ZEB1, which reduces cell–cell contact;^[^ ^35^, ^36^ ^]^ TANs‐secreted MMPs, serine proteases and cysteine cathepsins inhibit activation of NK cells and promote extravasation of circulating tumor cells;^[^ ^37^ ^]^ Moreover, TANs facilitate survival and seeding of metastatic cells by remodeling the extracellular matrix (ECM) and build an immunosuppressive environment.^[^ ^38^ ^]^

Neutrophils have recently been identified to exhibit procarcinogenic effects and predict poor prognosis in bladder cancer in bioinformatics and clinical researches.^[^ ^39^, ^40^ ^]^ However, the experimental evidence and the mechanism of how neutrophil promotes bladder cancer progression has not been fully uncovered, which is a barrier to identify neutrophil‐associated potential therapeutical target. Lu et al.^[^ ^41^ ^]^ reported that BCa cell derived CXCL5 to recruit and activate neutrophils, and then neutrophils secret VEGFC to promote lymphangiogenesis. Consistent with our findings, neutrophils do play a role in this BCa lymphatic metastasis. In addition, TANs sustain tumor angiogenesis through the release of the pro‐angiogenic factors BV8 and MMP9 that activate VEGFA.^[^ ^19^ ^]^ Furthermore, it is reported neutrophils recruited to sites of inflammation increase lymphangiogenesis by secreting VEGFA and VEGFD in the absence of B cells.^[^ ^26^ ^]^ However, whether TANs are involved in tumor lymphangiogenesis is unknown. We observed depletion of neutrophils significantly decreased footpad tumor lymphangiogenesis in BALB/c mice, which resulted in inhibited LN metastasis of BCa cells. Moreover, tumor‐derived CXCL1 and CXCL8 increased CXCR2, ERK and JNK pathways‐dependent production of VEGFA and MMP9 in neutrophils, which led to lymphangiogenesis. It is reported that neutrophil‐derived VEGFA stimulates lymphangiogenesis via macrophage‐secreted VEGFC and VEGFD.^[^ ^42^ ^]^ However, we observed neutrophils directly promoted the tube formation of HLECs, while tumor cells accelerated the progress. Moreover, neutralizing VEGFA by VEGFA‐specific neutralization antibody abolished the tube formation of HLECs. Thus, our results demonstrate that neutrophils directly regulate lymphangiogenesis via secreting VEGFA in parallel with VEGFC pathway.

ETV4 has been investigated to play a role in tumor metastasis and proliferation.^[^ ^43^, ^44^, ^45^ ^]^ Depletion of neutrophils diminished ETV4‐overexpression‐induced lymphangiogenesis in footpad tumor, which indicated ETV4 promoted lymphangiogenesis in a TAN‐dependent manner. However, depleting neutrophils in ETV4 overexpressed group did not completely inhibit ETV4 overexpression‐induced LN metastasis. Moreover, ETV4 also enhanced the migration and invasion abilities of BCa cells in vitro. Together, these results might indicate that ETV4 promoted LN metastasis depending on both TANs‐mediated lymphangiogenesis and tumor‐intrinsic migration and invasion. Moreover, using recent published BCa single‐cell sequencing data,^[^ ^46^ ^]^ we analyzed ETV4 expression at single‐cellular level (data not shown). ETV4 mainly expresses in epithelial cells (cancer cells). In addition, ETV4 also expresses in partial cancer‐associated fibroblasts (CAFs) and endothelial cells (ECs). While in immune cells, including B cells, T cells, mast cells, and myeloid cells, ETV4 was rarely expressed (data not shown). ETV4 has been reported to be fibroblast growth factor receptor (FGFR) targets, participating in embryonic development.^[^ ^45^, ^47^ ^]^ Blockade of FGF could inhibit growth of multiple types of cancer, partially due to regulation of ETV4 expression.^[^ ^48^ ^]^ However, the function of ETV4 in CAFs has not been reported. In endothelial cells, ETV4 act as an effector of Angiopoietin‐1 signaling to play significant angiogenic roles in physiologic status.^[^ ^49^ ^]^ Whether endothelial‐derived ETV4 participates in angiogenesis and lymphangiogenesis in cancer is worth to discover. Collectively, cancer cell is the major source of ETV4, at least in BCa, which promoted LN metastasis depending on both TANs‐mediated lymphangiogenesis and tumor‐intrinsic migration and invasion.

Since LN metastasis is a leading cause of BCa‐related mortality, intervention of LN metastasis might be an effective therapeutic strategy for improving patient prognosis. Meanwhile, lymphangiogenesis is an important part of LN metastasis. Here, we revealed that ETV4 promoted TANs‐mediated lymphangiogenesis and LN metastasis of BCa by regulating TANs infiltration. Moreover, high level expression of ETV4 was positively correlated with TANs and predicted high rates of LN metastasis and poor prognosis outcome of BCa. It is reported TANs are associated with metastasis in various cancers. However, directly targeting neutrophils in the patients might result in severe innate immune deficiency. Thus, ETV4 emerged as a potent target for inhibit TANs‐medicated lymphangiogenesis and LN metastasis of BCa.

Overall, our findings not only provide a novel mechanism of TANs associated LN metastasis of tumor cells via ETV4‐CXCL1/8‐CXCR2‐VEGFA/MMP9 pathway‐mediated lymphangiogenesis, but also point to potential targets for drug development against LN metastasis of BCa (Figure 7I).

## 4 Experimental Section

### Clinical Samples

All cancer tissue samples included in this study were pathologically diagnosed with bladder cancer between January 2004 and August 2019. Samples without clear pathological, clinical and survival information were excluded. A total of 207 (160 of them were used for survival analysis) formalin‐fixed, paraffin‐embedded BCa specimens termed Cohort 1, were obtained from patients undergoing surgery at the Sun Yat‐sen University Cancer Center (Guangzhou, Guangdong, China). Tissue microarrays containing 172 BCa specimens, termed Cohort 2, were purchased from Avila Biotechnology (Xi'an, Shaanxi, China). Meanwhile, 161 BCa specimens and 20 NAT, named Cohort 3, were acquired from the Sun Yat‐sen University Cancer Center (Guangzhou, Guangdong, China). Tissue cDNA containing 66 BCa and NAT, termed Cohort 4, were obtained from patients undergoing surgery at the Sun Yat‐sen Memorial Hospital (Guangzhou, Guangdong, China). Each sample was confirmed pathologically by two pathologists. The use of these specimens was approved by the Institutional Ethical Review Board for Research on the use of human subjects at Sun Yat‐sen Memorial Hospital (2019‐KY‐059).

### Immunohistochemistry (IHC)

IHC was performed as previously described.^[^ ^50^ ^]^ All tissue sections were dewaxed at 60 °C for 2 h followed by dimethylbenzene treatment. Different concentrations of ethanol were then used to hydration the tissues. Afterward, the antigen was repaired with EDTA, and the catalase blocked with 3% hydrogen peroxide. 5% BSA was used to block the sections, followed by incubation with the primary antibodies at 4°C overnight. Subsequently, the secondary antibodies were incubated, and DAB and hematoxylin were used to mark the antigen and counterstain the nuclei, respectively.

The CD66b^+^ or MPO^+^ or CD16^+^ or Ly6G^+^ or Gr1^+^cells were determined as the percentage of the positive signal in each field of view in the overall tissues by Image J. For quantification, CD66b^+^ or MPO^+^ or CD16^+^ or Ly6G^+^ or Gr1^+^cells were counted in at least 5 fields per section. The accuracy of automated measurements was confirmed by two pathologists. For ETV4 expression, two pathologists blindly quantified the expression of ETV4 in specimens according to a staining scoring system. Briefly, the proportion of positively stained cancer cells was assessed as a percentage. The intensity of immunostaining in each sample was graded as negative = 0, weak = 1, moderate = 2, or strong = 3. The staining score, termed H‐score, was then calculated as the numbers representing intensity multiples by the percentage of cells stained (H‐score = Intensity × percentage of positive cells). All specific antibodies are listed in Table S10 (Supporting Information).

### Immunofluorescence Staining

The dewaxing and hydration processes of the FFPE tissues were operated as previously mentioned.^[^ ^51^ ^]^ Antigen retrieval was performed using target retrieval solution, pH 9.0 in a pressure cooker for 15 to 20 min. Nonspecific binding was then blocked with 5% BSA for 30 min at room temperature. Cells for immunofluorescence were fixed with 4% paraformaldehyde for 30 min at room temperature, washed with PBS, and permeabilized with or without 0.2% Triton X‐100 in PBS for 20 min. Cells were then blocked in PBS with 2% BSA for 30 min at room temperature. Subsequently, the samples were incubated with mouse anti‐MPO (1:100), Rabbit anti‐homo‐LYVE1 (1:2000) and Rabbit anti‐mus‐LYVE1 (1:2000) overnight at 4 °C. The tissues were then incubated with fluor‐conjugated secondary antibodies in 1% BSA for 1 h at room temperature. Subsequentially, DAPI was then used for counterstaining the nuclei. Laser scanning confocal microscopy (Leica SP8 STED 3X, Germany) was applied to obtain the images. For quantification, MPO^+^, VEGFA^+^ or MMP9^+^ cells were counted in at least 5 fields per section. The accuracy of automated measurements was confirmed by two independent pathologists.

### Cell Culture

The human bladder cancer cell lines (UM‐UC‐3 and T24), HLEC and HEK293T cell line were obtained from the American Type Culture Collection (ATCC, Manassas, VA, USA). Roswell Park Memorial Institute (RPMI)‐1640 (Gibco, China) containing 10% fetal bovine serum (FBS) and 1% penicillin/streptomycin (Thermo Fisher Scientific, USA) were applied to culture T24 cells, ECM medium (Hyclone) containing 10% FBS and 1% penicillin/streptomycin applied to culture HLEC cells, whereas Dulbecco's modified Eagle medium (DMEM; Gibco, China) containing 10% FBS and 1% penicillin/streptomycin were applied to culture UM‐UC‐3 and HEK293T cells. All the cell lines were cultured in a humidified atmosphere of 5% CO_2_ at 37 °C. All cell lines used in this study were tested negative for mycoplasma contamination.

### RNA Isolation and qRT‐PCR

Total RNA was extracted from cells Trizol reagent (TaKaRa Biotechnology, China) according to the manufacturer's instruction. The quality of total RNA was assessed by Nanodrop 2000 (Thermo Fisher Scientific, USA). Total RNA was reverse transcribed with a PrimerScript RT‐PCR kit (TaKaRa Biotechnology, China). A standard SYBR Green PCR kit (Roche, Germany) protocol and LightCycler 480 real‐time instrument (Roche, Germany) were applied to perform the real‐time qPCR. The transcription levels of GAPDH were used as internal controls. The relative mRNA expression was calculated using the 2^−ΔΔCt^ method. All specific primers are listed in Table S11 (Supporting Information).

### RNA Interference

The siRNA oligonucleotides targeting the target protein and negative control siRNA were purchased from GenePharma (Shanghai, China) and are listed in Table S12 (Supporting Information). The siRNA transfections were performed according to the manufacturer's instructions and as previously described.^[^ ^52^ ^]^

### Western Blot

The cells were lysed using the protein extraction NP‐40 buffer (Beyotime, China) supplemented with a protease inhibitor cocktail and a phosphatase inhibitor. The protein concentration was measured with a BCA Protein Assay Kit (Thermo Fisher Scientific, USA). Total proteins were separated by SDS‐PAGE on 10% gels and transferred to 0.45 µm polyvinylidene fluoride (PVDF) membranes. Then, 5% skim milk (Beyotime, China) was used to block the membranes for 1 h at room temperature. Afterward, membranes were incubated with specific primary antibodies at 4°C overnight. After washing by tris buffer saline with Tween‐20 (TBST), the membranes were then incubated with HRP conjugated goat anti‐mouse or anti‐rabbit secondary antibodies (1:5000) accordingly and visualized using enhanced chemiluminescence. All specific antibodies are listed in Table S10 (Supporting Information).

### Plasmid Constructs

Mammalian expression plasmids for ETV4 and ETV4 mutants or reporter plasmids for CXCL1 and CXCL8 were constructed by standard molecular biology techniques. Sequence of CXCL1 or CXCL8 promoter and primer of ETV4 mutants were listed in Table S13 (Supporting Information).

### Transwell Assay

Cell invasion and migration assay were performed as previously mentioned. Briefly, Cells were suspended in 200 µLL serum‐free medium and seeded into the upper transwell chambers. A total 700 µL medium with 10% FBS was added into the lower chambers. After incubation at 37 °C, the cells in the upper chamber were softly removed by cotton swab. Then, the cell culture inserts were gently washed by PBS and immersed in paraformaldehyde for fixing the cells on the lower surface of the inserts. Finally, the cells were stained with 0.1% crystal violet for photographing and counting.

### HLECs Tube Formation Assay

Twenty‐four‐well plates were precoated with the Matrigel. HLECs were seeded into the 24‐well plates, and the neutrophil condition medium induced by corresponding UM‐UC‐3 or T24 cells was added to the wells. The resulting lymphatic tubes were fixed by paraformaldehyde and then photographed using an inverted microscope. The tube length was measured by Image J.

### Apoptosis Analysis

The assay was operated as previously mentioned.^[^ ^51^ ^]^ Briefly, the indicated cells were treated accordingly, then were collected and washed with PBS. The cells were stained by Annexin V‐FITC and PI to determine the percentage of apoptosis in a flow cytometer (Beckman cytoFLEX, USA).

### Enzyme‐Linked Immunosorbent Assay (ELISA)

The CXCL1 (4Abio, CHE0065) and CXCL8 (4Abio, CHE0011) protein secreted to the medium of the T24 and UM‐UC‐3 cells or the VEGFA (4Abio, CHE0043) and MMP9 (Proteintech, KE00164) protein secreted to the medium of the neutrophils were quantified by ELISA kits. Briefly, the culture medium was collected and centrifuged at 1000 g for 5 min to remove the residual cells. The supernatant was diluted to different concentrations, and was added to the coated wells, then washed and harvested with the secondary antibody. Afterwards, the secondary antibody was discarded, and substrate solution was added, harvest according to the manufacturer's instructions, then the assay was terminated by the stop solution. The absorbance OD at 450 nm was determined by a microplate reader.

### Lentivirus Transduction

To establish the stable overexpression cell lines, the full‐length ETV4 with or without point mutations, or shRNA sequences were cloned into the pCDH‐CMV‐MCS‐EF1‐Puro or pLKO.1‐Puro vectors, which were co‐transfected into HEK‐293T cells with psPAX2 and pMD2.G plasmids. Lentiviral production and infection were performed as previously described.^[^ ^53^ ^]^

### Popliteal Lymph Node Metastasis Model

The BALB/c nude mice (4‐5 weeks old) were obtained from the Experimental Animal Center, Sun Yat‐sen University (Guangzhou, China). The animal experiment procedures were approved by the Institutional Animal Care and Use Committee of Sun Yat‐sen University (SYSU‐IACUC‐2022‐B1505). The footpads of mice were inoculated with 100 µL PBS suspensions of UM‐UC‐3/luc cells (1 × 10^7^)that were transduced with full‐length ETV4 or shRNA of ETV4. Intraperitoneal injection with the IgG negative control or the Ly6G neutralizing antibody (300 µg per mouse) to deplete neutrophils as shown in Figure 2a. Lymph node metastasis was imaged with bioluminescence imaging system (PerkinElmer, IVIS Spectrum Imaging System). The primary tumors and popliteal lymph nodes were enucleated and captured. Afterwards, the tissues were digested to obtain the single‐cell suspension for further flow cytometry analyses to quantify the specific populations of the neutrophils.

### Co‐Immunoprecipitation (Co‐IP)

Co‐immunoprecipitation was performed as the previous study.^[^ ^51^ ^]^ Briefly, cells were lysed in IP lysis buffer with protease inhibitor cocktail (Thermo Fisher Scientific, USA). Lysates were incubated with the indicated antibodies at 4 °Covernight. 40 µL of Dynabeads Protein A (Thermo Fisher Scientific, USA) was then added to the lysates to incubate for 2.5 h at room temperature. The beads–protein complex was washed 3 times with the prelysis buffer, eluted with 1× loading buffer and boiled for 10 min at 95 °C followed by Western blotting.

### ChIP

ChIP was conducted according to the manufacturer's instructions and as previously reported.^[^ ^54^ ^]^ The indicated cells were treated with 1% formaldehyde for 10 min and lysed with membrane extraction buffer containing protease/phosphatase inhibitors after washing. After concentrating, the pellets were digested by MNase at 37 °C for 15 min. After incubating with MNase stop solution on ice for 5 min and concentrating, the pellets were resuspended in IP dilution buffer and sonicated. After concentrating, the supernatants were proceeded to the immunoprecipitation with the appropriate antibody (anti‐IgG or anti‐ETV4). After washing with low salt, high salt and LiCl buffer, elution buffer was used to harvest the chromatin fragments. Finally, decrosslinking was performed, and the enrichment was examined using qPCR. The primers for ChIP‐qPCR are listed in Table S11 (Supporting Information).

### Cytosolic and Nucleus Fraction Isolation

Cell cytoplasm and nucleus fraction isolation was conducted according to the manufacturer's protocol (Pierce, Rockford, USA). Briefly, cells were collected and washed twice with cold PBS. Then the cell suspension was centrifuged at 500 g for 3 min. The cell pellet was suspended in 200 µL of cytoplasmic extraction reagent I by vortexing, and incubated on ice for 10 min. Afterwards, 11 µL of a second cytoplasmic extraction reagent II was added, vortexed for 5 s, incubated on ice for 1 min and centrifuged for 5 min at 16 000 g. The supernatant fraction (cytoplasmic extract) was transferred to a pre‐chilled tube. The remaining pellet fraction, which contains crude nuclei, was resuspended in 100 µL of nuclear extraction reagent by vortexing during 15 s and incubated on ice for 10 min, then centrifuged for 10 min at 16 000 g. The resulting supernatant, constituting the nuclear extract, was used for the subsequent experiments.

### Flow Cytometry

Cells separated from peripheral blood or single‐cell suspension of tissues were treated by ACK lysis buffer to eliminate the residual red blood cells, and then resuspended in PBS. Florence‐conjugated antibodies were used to stain the corresponding marker. All cells were analyzed using flow cytometer (Beckman cytoFLEX, USA). All specific antibodies are listed in Table S10 (Supporting Information).

### Neutrophil Isolation

Neutrophils were isolated from peripheral blood of healthy donors by density gradient separation using Lymphoprep (STEMCELL, 07851) and centrifuging at 400 g for 20 min. The purity of the isolated neutrophils was determined by flow cytometry.

### Neutrophil Migration Assays

Freshly isolated neutrophils suspended in 200 µL RPMI 1640 were added into the upper chambers, and a total 400 µL mixture of RPMI 1640 and UM‐UC‐3 CM was added to the lower chambers. The attracted neutrophils were counted after 2 h.

### Statistical Analysis

All the statistical graphs and corresponding statistical analyses were performed using GraphPad Prism 7 software, and error bars indicate S.E.M. or S.D. The statistical significance of differences between groups was determined by unpaired/paired Student's t tests or one‐way ANOVA. Spearman's correlation analysis was performed to determine the correlation between two variables. Kaplan‐Meier method and log‐rank test were used for the survival data. A multivariate Cox proportional hazards model was used to estimate the adjusted hazard ratios (HRs) and 95% confidence intervals and identify independent prognostic factors. The optimal cutoff value was used to define the gene expression level for all survival analyses in the study. All the other statistical analyses in the present study were performed using R (version 4.1.0). A *p* value of <0.05 was considered statistically significant.

## Conflict of Interest

The authors declare no conflict of interest.

## Author Contributions

Q.Z., S.L., and H.W. contributed equally to this work. Q.Z., X.C. and T.‐X.L. designed the study. Q.Z., S.L., H.‐J.W., K.‐H.X., S.‐T.C., M.H., and R.‐H.X. performed the experiments and analysis. Q.Z., X.C., S.L., and T.‐X.L. wrote and reviewed the paper. All the authors analyzed data.

## Supporting information

Supporting Information Figures

Supporting Information Tables

## Data Availability

The data that support the findings of this study are available from the corresponding author upon reasonable request.
