# Correction to “ETV4 Mediated Tumor‐Associated Neutrophil Infiltration Facilitates Lymphangiogenesis and Lymphatic Metastasis of Bladder Cancer”

**DOI:** 10.1002/advs.73278

**Published:** 2025-12-16

Qiang Zhang, Sen Liu, Hongjin Wang, Kanghua Xiao, Junlin Lu, Siting Chen, Ming Huang, Ruihui Xie, Tianxin Lin, Xu Chen

Adv Sci (Weinh).2023Apr;10(11):e2205613. https://doi.org/10.1002/advs.202205613.

Original Figure 6A:


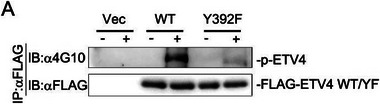


In Figure 6 of the original publication, the panel A: FLAG‐ETV4 WT/YF blot was incorrectly inserted. The correct image of Figure 6A should be as follows:


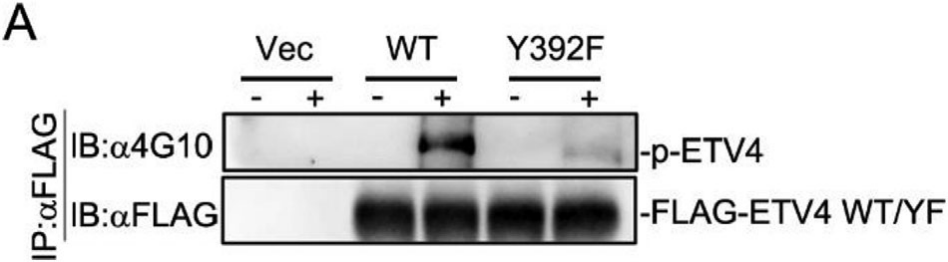


Original Figure S9C (Supporting Information):


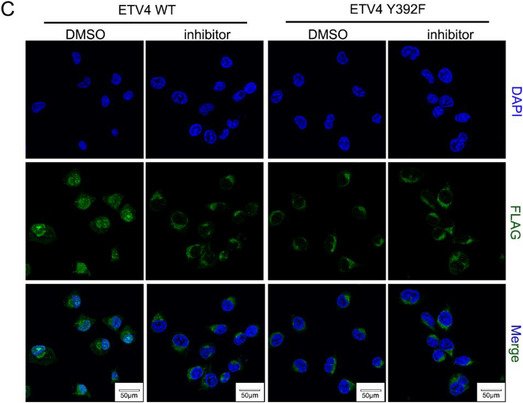


In Figure S9 (Supporting Information) of the original publication, panel C: immunofluorescence images of the “ETV4 WT” group were incorrect. The correct image of Figure S9C (Supporting Information) should be as follows:


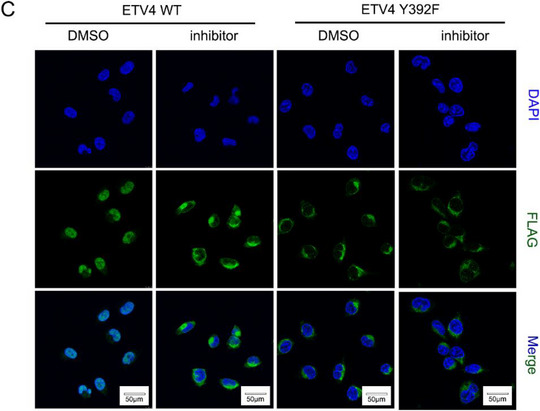


These errors occurred solely during the final stages of image compilation and layout, representing a technical oversight, and not because of data fabrication or falsification.

We apologize for the errors.

