# Supplementary material for: ETV4 Mediated Tumor‐Associated Neutrophil Infiltration Facilitates Lymphangiogenesis and Lymphatic Metastasis of Bladder Cancer
Source: Adv Sci (Weinh). 2023 Jan 20;10(11):2205613. doi: 10.1002/advs.202205613 (PMC10104629; doi:10.1002/advs.202205613)
Supplement: Supplementary file 1 — Supporting Information Figures [file ADVS-10-2205613-s001.pdf]

ADVANCED SCIENCE
Open Access

Supporting Information

for *Adv. Sci.*, DOI 10.1002/advs.202205613

ETV4 Mediated Tumor-Associated Neutrophil Infiltration Facilitates Lymphangiogenesis and Lymphatic Metastasis of Bladder Cancer

Qiang Zhang, Sen Liu, Hongjin Wang, Kanghua Xiao, Junlin Lu, Siting Chen, Ming Huang, Ruihui Xie, Tianxin Lin and Xu Chen**

Supplementary Figures

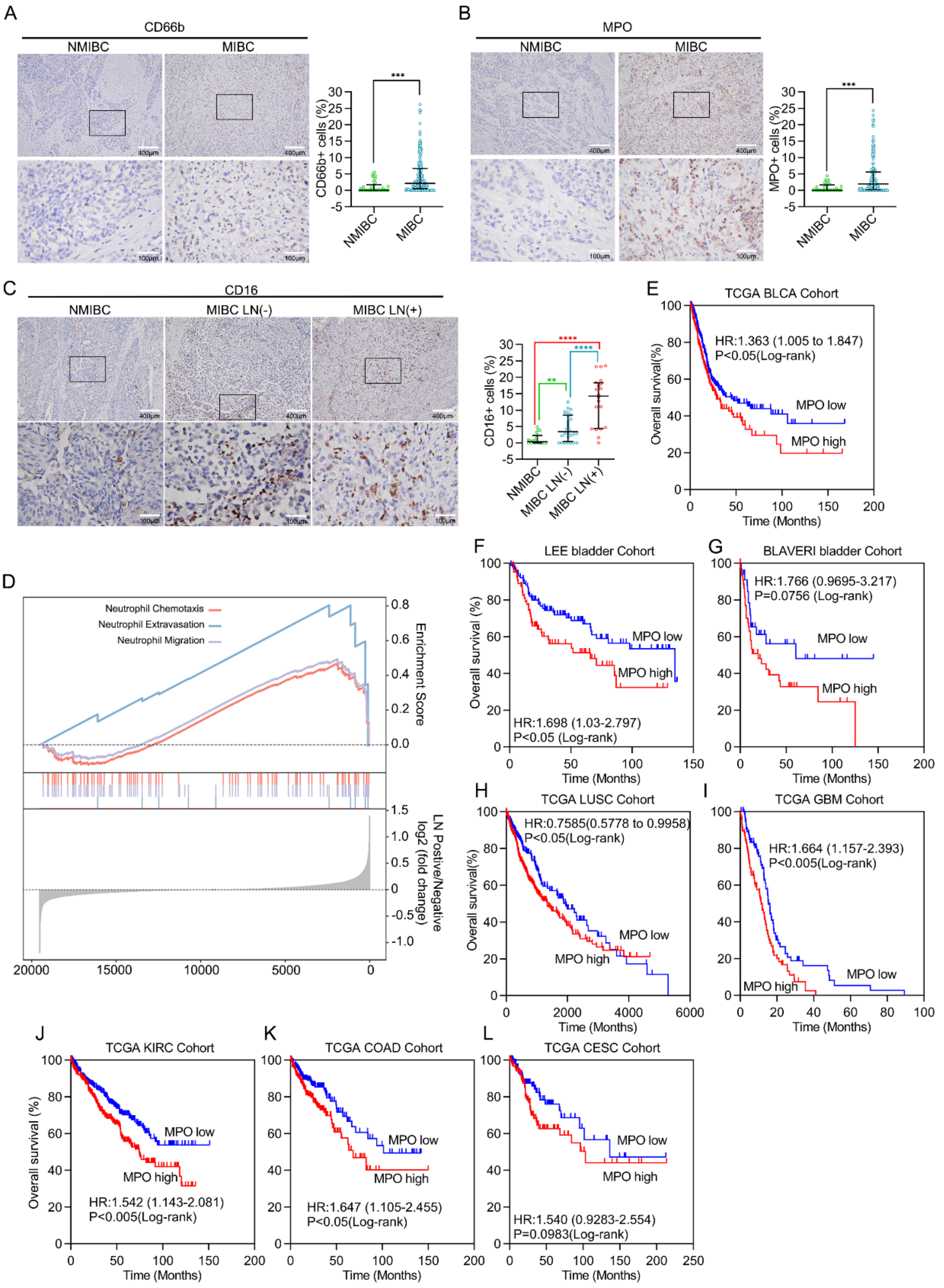

Figure S1. TANs predict poor prognosis in multiple cancers.

(**A, B**) Representative IHC images straining for CD66b (**A**) and MPO (**B**) (left) and quantification of CD66b^{+} (**A**) and MPO^{+} (**B**) cells infiltrated in NMIBC and MIBC (right). (**C**) Representative IHC images straining for CD16^{+} and quantification of CD16^{+} cells infiltrated in

NMIBC (left), MIBC LN(-) (mid), and MIBC LN(+) (right). **(D)** Gene-set enrichment analysis (GSEA) of neutrophil chemotaxis, extravasation and migration pathways in LN (-) and LN (+) BCa tissues from TCGA database. (**E-G**) Kaplan-Meier survival analysis of OS of BCa patients with high level versus low level MPO expression in TCGA cohort (242 vs 160) (**E**), LEE bladder Cohort (64 vs 101) (**F**) or BLAVERI bladder Cohort (65 vs 34) (**G**) from Oncomine database. (**H-L**) Kaplan-Meier survival analysis of OS of patients with high level versus low level MPO expression in Lung Squamous Cell Carcinoma (LUSC, 211 vs 277) **(H)**, Glioblastoma (GBM, 76 vs 76) **(I),** Kidney Clear Cell Carcinoma (KIRC, 277 vs 245) (**J**), Colon Cancer (COAD, 230 vs 210) (**K**), and Esophageal Cancer (CESC, 137 vs 127) (**L**) from TCGA database. Statistical significance was assessed using two-tailed t tests or one-way ANOVA. *** $p < 0.001$.

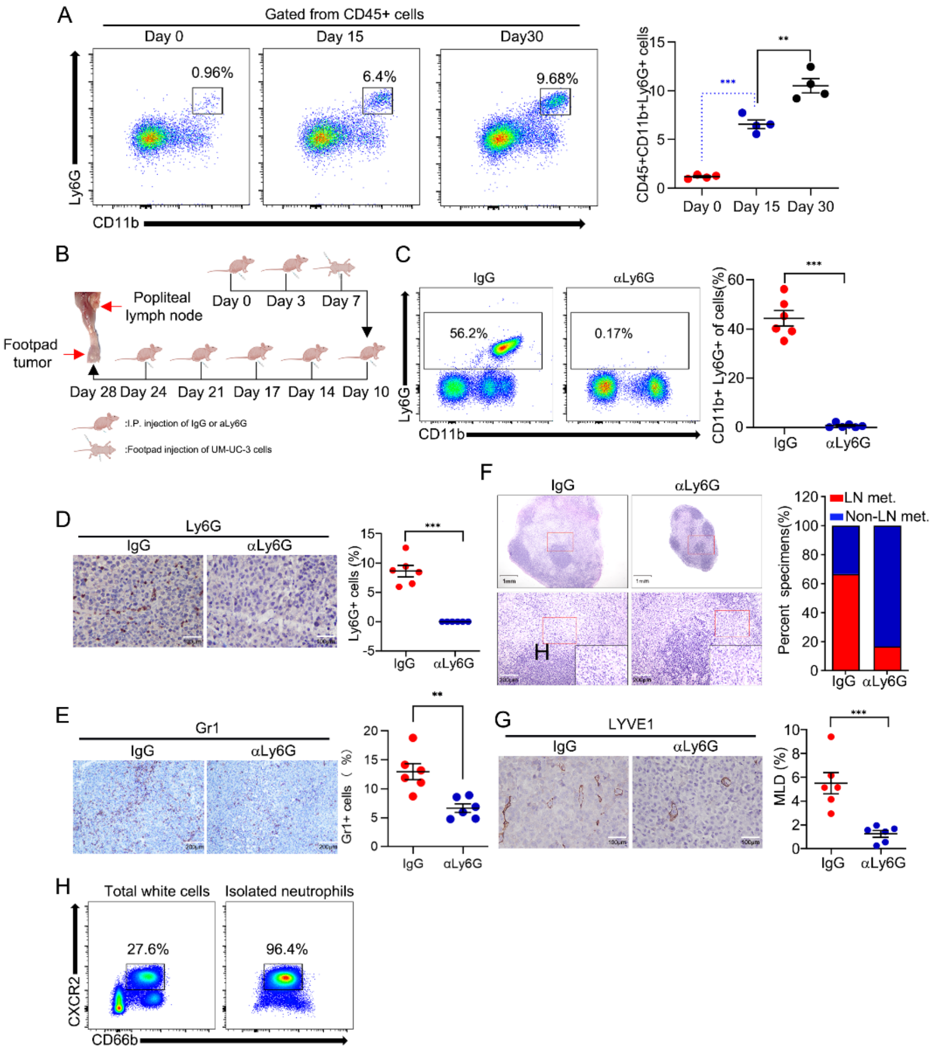

Figure S2. TANs promote LN metastasis of BCa in mice.

(**A**) Flow cytometry analysis of $CD45^{+}CD11b^{+}Ly6G^{+}$ neutrophils from fresh surgically excised footpad tumor tissues. (**B**) The strategy of neutrophil depletion and LN metastasis model establishment in node mice. (**C**) Flow cytometry analysis of $CD11b^{+}Ly6G^{+}$ neutrophils in peripheral blood. (**D**) Representative IHC images straining for Ly6G (left) and quantification of $Ly6G^{+}$cells infiltrated in indicated footpad tumor (right). (**E**) Representative IHC images straining for Gr1 (left) and quantification of $Gr1^{+}$cells infiltrated in indicated footpad tumor (right). **(F)** Representative H&E images (right) and quantification (right) of LN metastasis status in indicated LNs. **(G)** Representative IHC images straining for LYVE1 (left) and quantification of $LYVE1^{+}$cells in indicated footpad tumor (right). (**H**) Flow cytometry analysis of $CD66b^{+}CXCR2^{+}$ cells in total white cells and isolated neutrophils. Statistical significance was assessed using two-tailed t tests or one-way ANOVA. ** $p < 0.01$, ***$p < 0.001$.

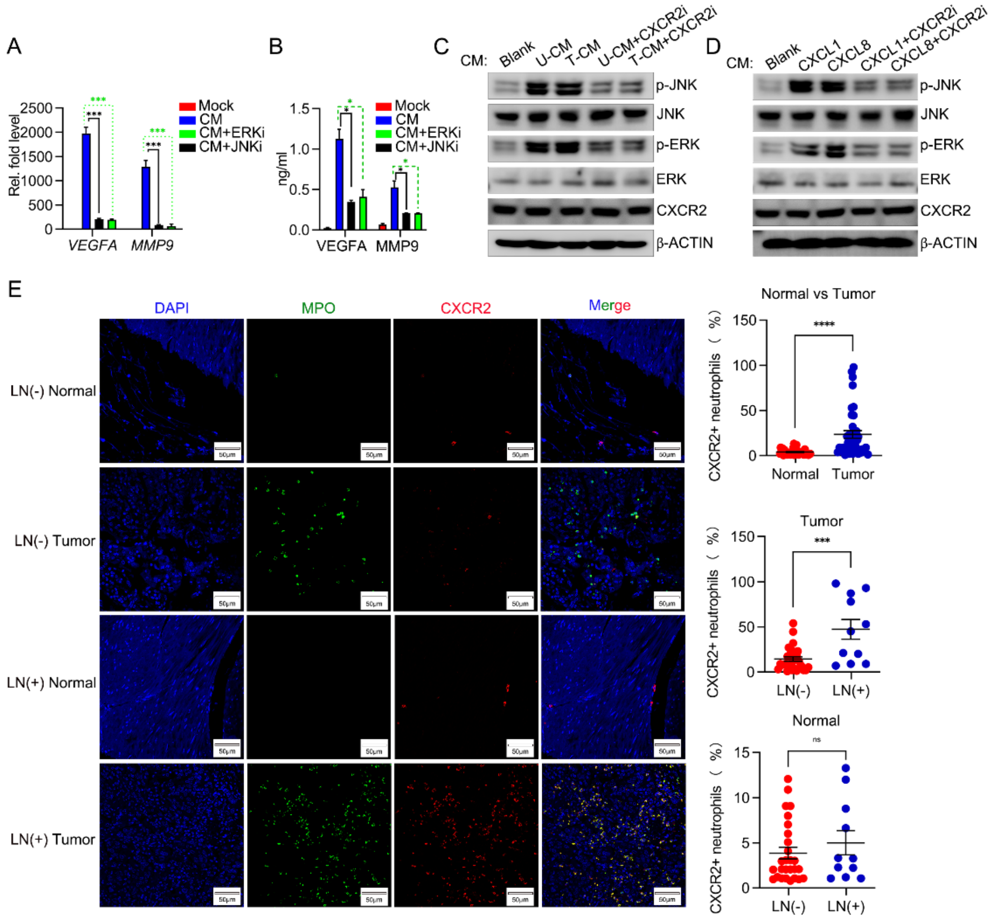

Figure S3. CXCL1/8-CXCR2 activate ERK-JNK signaling in TANs

(A) qRT-PCR analysis of VEGFA and MMP9 mRNA in neutrophils treated with PBS, UM-UC-3 cells Culture Media (CM), and CM combined with inhibitor of ERK (trameitinib, 10 μM) or JNK (SP600125, 10 mM) for 3 h, respectively. **(B)** ELISA analysis of VEGFA and MMP9 in the supernatants of neutrophils treated with above treatment for 12 h. **(C)** Immunoblot analysis of p-JNK, JNK, p-ERK, ERK, CXCR2 and β-Actin in neutrophils treated with UM-UC-3 or T24 CM for 3 h. **(D)** Immunoblot analysis of p-JNK, JNK, p-ERK, ERK, CXCR2 and β-Actin in neutrophils treated with the indicated supernatant for 3 h, respectively. **(E)** Representative immunofluorescence images (left) staining for MPO and CXCR2 in normal and tumor tissue from patients with or without lymph node metastasis and the quantification (right) of $CXCR2^{+}$ neutrophils between the indicated groups.

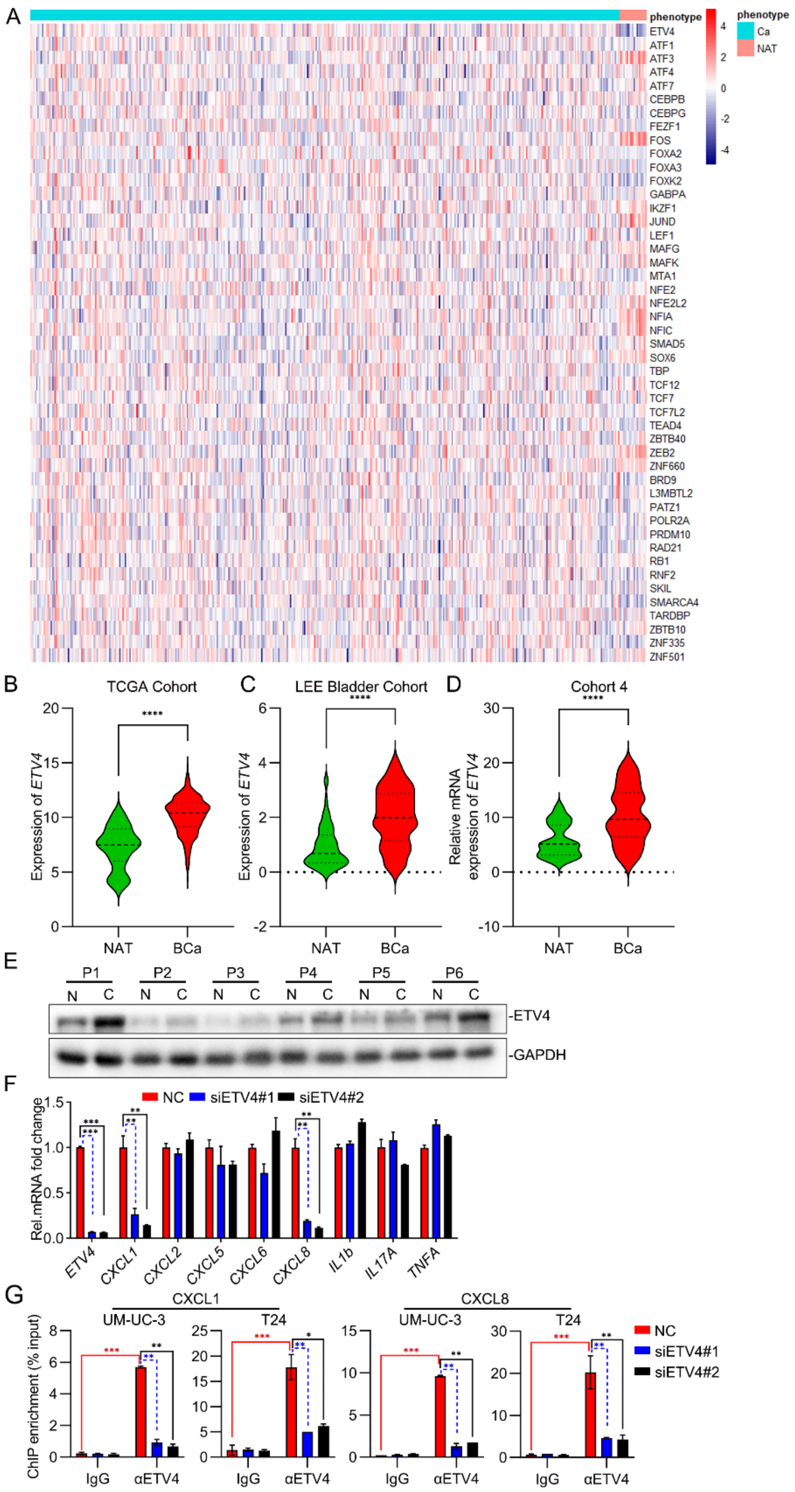

Figure S4. ETV4 is overexpressed in BCa.

(**A**) Heatmap of expression of the indicated genes from TCGA database of BCa. (**B-C**) Analysis of the expression of ETV4 in TCGA database of BCa (**B**) and LEE bladder Cohort (**C**). (**D**) qRT-PCR analysis of *ETV4* mRNA in Cohort 4. (**E**) Immunoblot analysis of ETV4 and GAPDH in NAT (N) or BCa (C) tissues. (**F**) qRT-PCR analysis of *ETV4*, *CXCL1*, *CXCL5*, *CXCL8*, *IL1b*, *IL17A* and *TNFA* mRNA in UM-UC-3 cells treated with NC, siETV4#1 or siETV4#2 for 48 h.

(G) Values normalized to input of ChIP analysis related to Fig 3F. Statistical significance was assessed using two-tailed t tests or one-way ANOVA. $^{**}p < 0.01$, $^{***}p < 0.001$, $^{****}p < 0.0001$. Data are representative of two (**E**) or three independent experiments (mean ± S.D. in **F**).

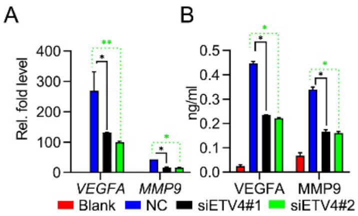

Figure S5. Tumor ETV4 promotes VEGFA and MMP9 expression in TANs.

(**A**) qRT-PCR analysis of *VEGFA* and *MMP9* mRNA in neutrophils incubated with UM-UC-3 CM. (**B**) ELISA analysis of VEGFA and MMP9 in the supernatants of neutrophils treated with the indicated supernatant for 12 h. Statistical significance was assessed using two-tailed t tests or one-way ANOVA. *p < 0.05, **p < 0.01. Data are representative of two (**B**) or three independent experiments (mean ± S.D. in **A**).

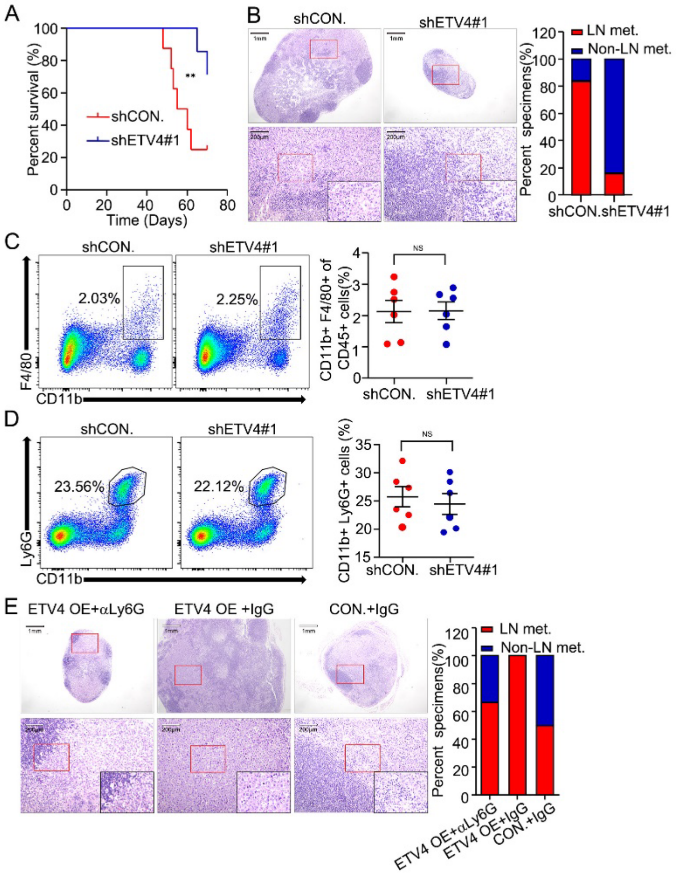

Figure S6. ETV4 promotes LN metastasis of BCa in mice.

(**A**) Kaplan-Meier survival analysis of mice inoculated with shCON. and shETV4#1 UM-UC-3 cells. (**B**) Representative H&E images (left) and quantification (right) of LN metastasis status in indicated LNs. (**C**) Flow cytometry analysis of $CD11b^+F4/80^+$ cells in indicated footpad tumors. (**D**) Flow cytometry analysis of $CD11b^+Ly6G^+$ neutrophils in spleen. (**E**) Representative H&E images (left) and quantification (right) of LN metastasis status in LNs from the empty vector (CON.) or ETV4 overexpression vector stably transfected UM-UC-3 cells and IgG or neutralizing antibody of Ly6G. Statistical significance was assessed using two-tailed t tests or one-way ANOVA. $^{**}p < 0.01$.

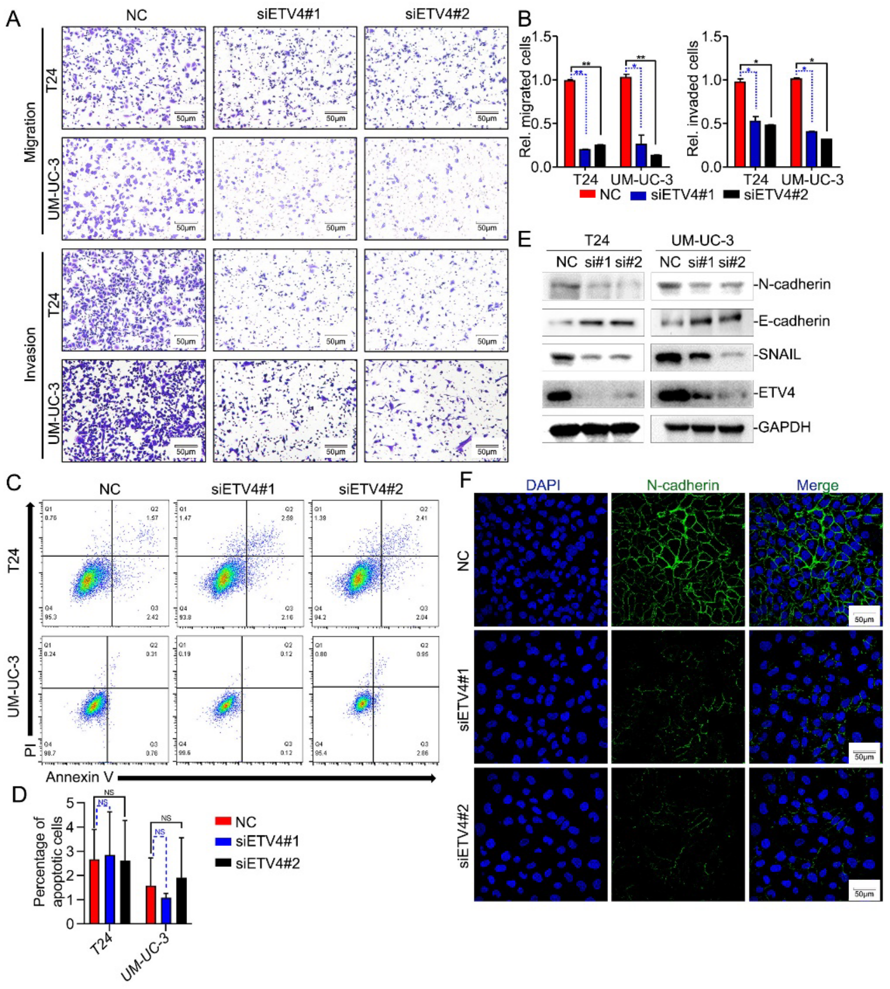

Figure S7. ETV4 promotes migration and invasion of BCa cells in vitro.

(**A, B**) Representative images (**A**) and analysis (**B**) of transwell migration and invasion in T24 and UM-UC-3 cells treated with NC, siETV4#1 or siETV4#2. (**C, D**) Flow cytometry analysis of Annexin V and PI staining T24 and UM-UC-3 cells treated as indicated. (**E**) Immunoblot analysis of N-cadherin, E-cadherin, SNAIL, ETV4 and GAPDH in T24 and UM-UC-3 cells. (**F**) Representative immunofluorescence images staining for N-cadherin in T24 cells treated as indicated. Statistical significance was assessed using two-tailed t tests or one-way ANOVA. *p <0.05, **p < 0.01. Data are representative of two (**E, F**) or three independent experiments (mean ± S.D. in **A-D**).

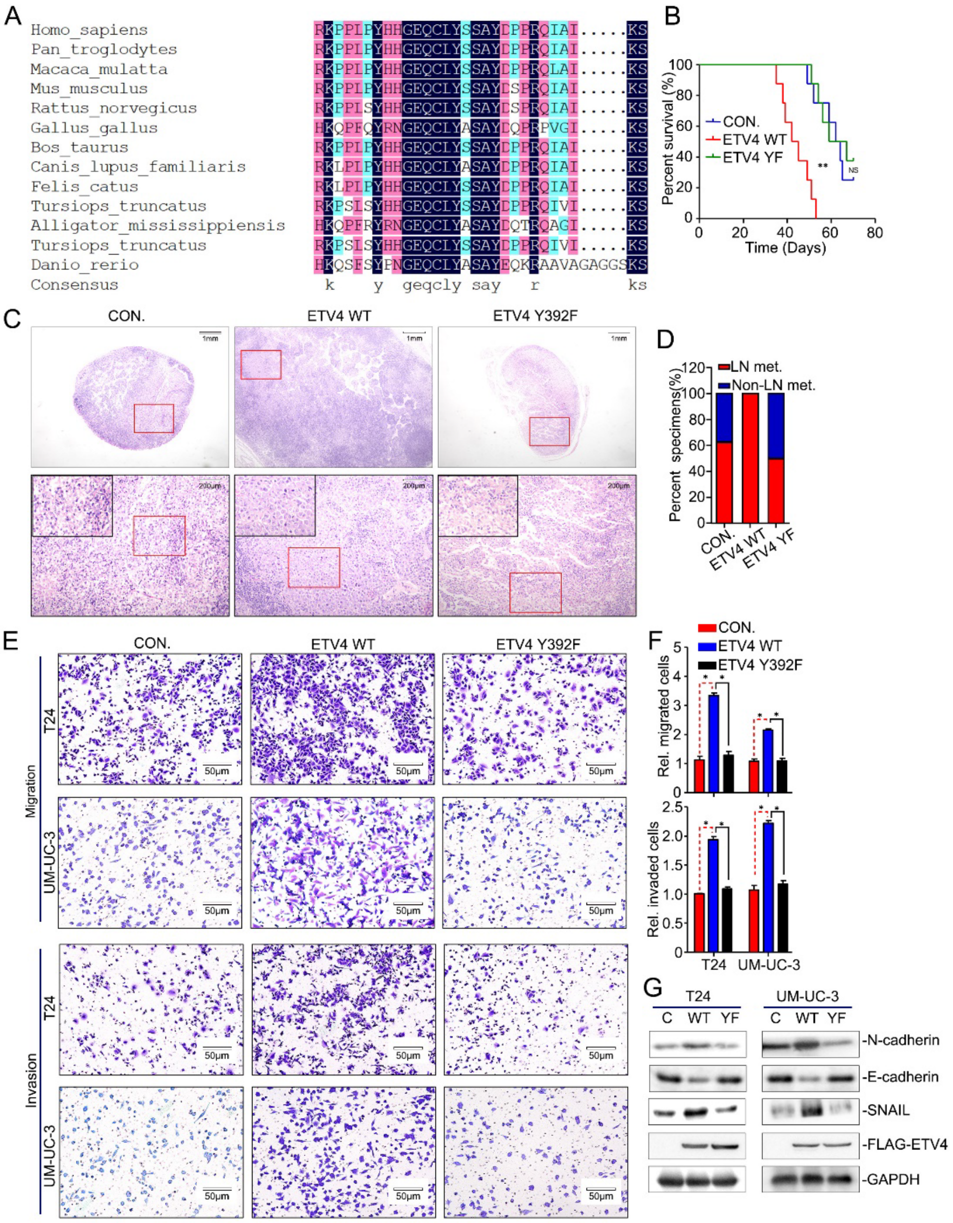

Figure S8. Y392 of ETV4 is required for the pro-LN-metastatic effect.

(**A**) Alignment of primary sequences of Homo. sapiens ETV4 and its homologues in 11 species: Pan troglodytes, Macaca mulatta, Mus musculus, Rattus norvegicus, Gorilla gorilla, Bos taurus, Canis familiaris, Felis catus, Tursiops truncates, Alligator mississippiensis and Danio rerio. Consensus sequences are listed at the left of the column; conserved amino acids are highlighted in black. (**B**) Kaplan-Meier survival analysis of mice inoculated with CON and ETV4 WT or Y392F overexpression vector stably transfected UM-UC-3 cells. (**C, D**) Representative H&E images (**C**) and quantification (**D**) of LN metastasis status in indicated LNs. (**E, F**) Representative images (**E**) and analysis (**F**) of transwell migration and invasion in the empty vector (CON.) or ETV4 WT or Y392F overexpression vector stably transfected T24 and UM-UC-3 cells. **(G)** Immunoblot analysis of N-cadherin, E-cadherin, SNAIL, ETV4 and GAPDH in in the empty vector (C) or ETV4 WT or Y392F (YF) overexpression vector stably transfected T24 and UM-UC-3 cells. Statistical significance was assessed using two-tailed t tests or one-

way ANOVA. **$p < 0.01$.

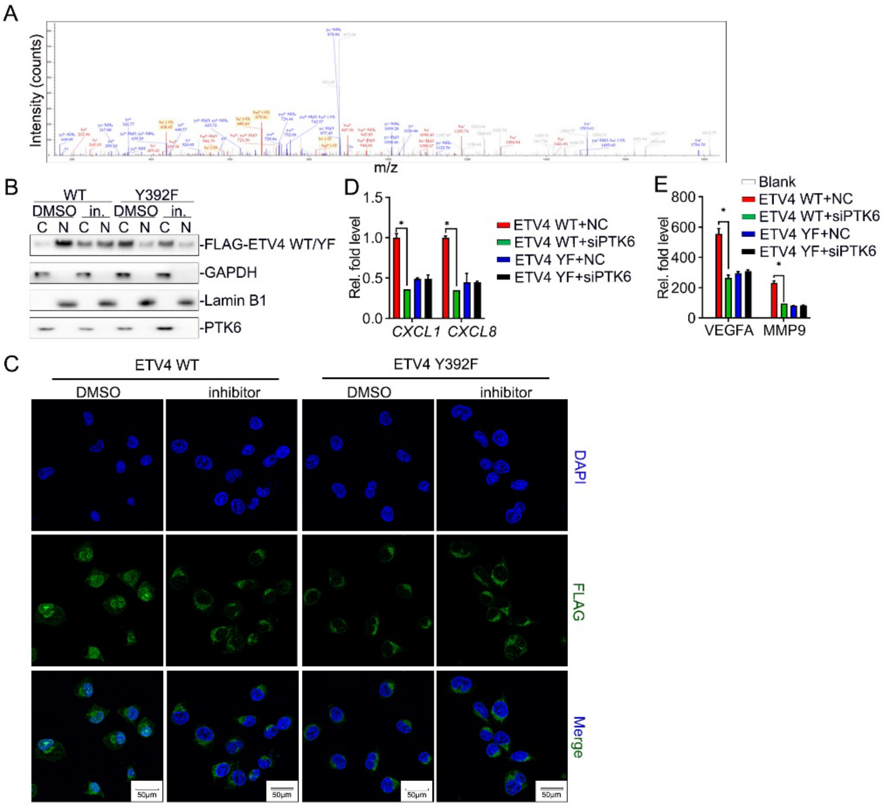

Figure S9. Inhibition of PTK6 suppressed the nuclear translocation of ETV4.

(**A**) Mass spectrometry (MS) identification of cytoplasmic ETV4-interacting proteins. (**B**) Immunoblot analysis of cytoplasmic and nuclear FLAG-ETV4, PTK6, GAPDH and Lamin B1 in UM-UC-3 cells that stably express FLAG-ETV4 WT or Y392F, treated with DMSO or inhibitor of PTK6 (Tilfrinib, 20 μM) for 36 h. (**C**) Representative immunofluorescence images staining for FLAG-ETV4 in UM-UC-3 cells treated as indicated. (**D**) qRT-PCR analysis of *CXCL1* and *CXCL8* mRNA in UM-UC-3 cells treated as indicated. (**E**) qRT-PCR analysis of *VEGFA* and *MMP9* mRNA in neutrophils incubated with indicated UM-UC-3 CM. Statistical significance was assessed using two-tailed t tests. *p <0.05. Data are representative of two (**A-C**) or three independent experiments (mean ± S.D. in **D, E**).
