# Supplementary material for: ETV4 Mediated Tumor‐Associated Neutrophil Infiltration Facilitates Lymphangiogenesis and Lymphatic Metastasis of Bladder Cancer
Source: Adv Sci (Weinh). 2023 Jan 20;10(11):2205613. doi: 10.1002/advs.202205613 (PMC10104629; doi:10.1002/advs.202205613)
Supplement: Supplementary file 2 — Supporting Information Tables [file ADVS-10-2205613-s002.pdf]

ADVANCED SCIENCE
Open Access

Supporting Information

for *Adv. Sci.*, DOI 10.1002/advs.202205613

ETV4 Mediated Tumor-Associated Neutrophil Infiltration Facilitates Lymphangiogenesis and Lymphatic Metastasis of Bladder Cancer

Qiang Zhang, Sen Liu, Hongjin Wang, Kanghua Xiao, Junlin Lu, Siting Chen, Ming Huang, Ruihui Xie, Tianxin Lin and Xu Chen**

Supplemental Table 1. Correlations between CD66b expression levels and clinicopathological characteristics in 160 bladder cancer cases from Cohort 1.

Characteristics	Cases	CD66b expression		*P*
		Low (%)	High (%)	
Total	160	84(52.5)	76(47.5)	
Age(years)				0.771
<65	96	49 (58.3)	47 (61.8)	
≥65	64	35 (41.7)	29 (38.2)	
Gender				0.226
Female	19	7 (8.3)	12 (15.8)	
Male	141	77 (91.7)	64 (84.2)	
Smoke				0.464
No	72	35 (41.7)	37 (48.7)	
Yes	88	49 (58.3)	39 (51.3)	
Tumor number				**0.005**
1	61	23 (27.4)	38 (50.0)	
≥2	99	61 (72.6)	38 (50.0)	
Tumor size(cm)				0.709
<3	73	40 (47.6)	33 (43.4)	
≥3	87	44 (52.4)	43 (56.6)	
Histological grade				**0.020**
Low	58	38 (45.2)	20 (26.3)	
High	102	46 (54.8)	56 (73.7)	
T stage				**0.012**
pTa-pT1	26	20 (23.8)	6 (7.9)	
pT2-pT4	134	64 (76.2)	70 (92.1)	
N stage				**<0.001**
pN0	110	70 (83.3)	40 (52.6)	
pN1-3	50	14 (16.7)	36 (47.4)	
Alive status				**0.001**
No	94	60 (71.4)	34 (44.7)	
Yes	66	24 (28.6)	42 (55.3)	

Significant *P* values are shown in bold.

Supplemental Table 2. Correlations between MPO expression levels and clinicopathological characteristics in 160 bladder cancer cases from Cohort 1.

Characteristics	Cases	MPO expression		*P*
		Low (%)	High (%)	
Total	160	89(55.6)	71(44.4)	
Age(years)				0.111
<65	96	48 (53.9)	48 (67.6)	
≥65	64	41 (46.1)	23 (32.4)	
Gender				0.309
Female	19	8 (9.0)	11 (15.5)	
Male	141	81 (91.0)	60 (84.5)	
Smoke				0.620
No	72	38 (42.7)	34 (47.9)	
Yes	88	51 (57.3)	37 (52.1)	
Tumor number				**0.006**
1	61	25 (28.1)	36 (50.7)	
≥2	99	64 (71.9)	35 (49.3)	
Tumor size(cm)				0.775
<3	73	42 (47.2)	31 (43.7)	
≥3	87	47 (52.8)	40 (56.3)	
Histological grade				0.083
Low	58	38 (42.7)	20 (28.2)	
High	102	51 (57.3)	51 (71.8)	
T stage				**0.002**
Ta-T1	26	22 (24.7)	4 (5.6)	
T2-T4	134	67 (75.3)	67 (94.4)	
N stage				**<0.001**
N0	110	76 (85.4)	34 (47.9)	
N1-3	50	13 (14.6)	37 (52.1)	
Alive status				**0.008**
Yes	94	61 (68.5)	33 (46.5)	
No	66	28 (31.5)	38 (53.5)	

Significant *P* values are shown in bold.

Supplemental Table 3. Univariate and multivariate analysis of CD66b expression and clinicopathological factors associated with overall survival (OS) in bladder cancer from Cohort 1.

Prognostic parameters	Univariate analysis		Multivariate analysis*	
	HR (95% CI)	*P*	HR (95% CI)	*P*
Age (years) (≥65 vs. < 65)	1.46(0.90-2.37)	0.130	-	-
Gender (Male vs Female)	0.97(0.46-2.03)	0.932	-	-
Smoke (Yes vs. No)	1.03(0.63-1.68)	0.902	-	-
Tumor size (cm) (≥3 vs. < 3)	0.99(1.01-0.61)	0.964	-	-
Tumor number (≥2 vs. 1)	0.77(0.47-1.25)	0.291	-	-
Histological stage (High vs. Low)	1.65(0.97-2.79)	0.064	-	-
T stage (T2-T4 vs. Ta-T1)	1.61(0.77-3.37)	0.210	-	-
N stage (N1-3 vs. N0)	2.67(1.58-4.48)	**<0.001**	2.07(1.21-3.54)	**0.008**
CD66b expression (High vs. Low)	2.78(1.68-4.61)	**<0.001**	2.35(1.39-3.98)	**0.001**

*Variables significantly associated with OS by univariate analysis were adopted as covariates in multivariable analysis. Significant *P* values are shown in bold. HR > 1, risk for death increased; HR < 1, risk for death reduced.

Supplemental Table 4. Univariate and multivariate analysis of MPO expression and clinicopathological factors associated with overall survival (OS) in bladder cancer from Cohort 1.

Prognostic parameters	Univariate analysis		Multivariate analysis*	
	HR (95% CI)	*P*	HR (95% CI)	*P*
Age (years) (≥65 vs. < 65)	1.46(0.90-2.37)	0.130	-	-
Gender (Male vs. Female)	0.97(0.46-2.03)	0.932	-	-
Smoke (Yes vs. No)	1.03(0.63-1.68)	0.902	-	-
Tumor size (cm) (≥3 vs. < 3)	0.99(1.01-0.61)	0.964	-	-
Tumor number (≥2 vs. 1)	0.77(0.47-1.25)	0.291	-	-
Histological stage (High vs. Low)	1.65(0.97-2.79)	0.064	-	-
T stage (T2-T4 vs. Ta-T1)	1.61(0.77-3.37)	0.210	-	-
N stage (N1-3 vs. N0)	2.67(1.58-4.48)	**<0.001**	2.08(1.19-3.65)	**0.011**
MPO expression (High vs. Low)	2.28(1.40-3.74)	**0.001**	1.80(1.05-3.07)	**0.032**

*Variables significantly associated with OS by univariate analysis were adopted as covariates in multivariable analysis. Significant *P* values are shown in bold. HR > 1, risk for death increased; HR < 1, risk for death reduced.

Supplemental Table 5. Univariate and multivariate analysis of CD66b expression and clinicopathological factors associated with disease-free survival (DFS) in bladder cancer from Cohort 1.

Prognostic parameters	Univariate analysis		Multivariate analysis*	
	HR (95% CI)	*P*	HR (95% CI)	*P*
Age (years) (≥65 vs. < 65)	1.19(0.74-1.90)	0.472	-	-
Gender (Male vs Female)	1.11(0.53-2.32)	0.777	-	-
Smoke (Yes vs. No)	1.19(0.75-1.89)	0.464	-	-
Tumor size (cm) (≥3 vs. < 3)	0.89(0.56-1.41)	0.617	-	-
Tumor number (≥2 vs. 1)	0.76(0.47-1.22)	0.249	-	-
Histological stage (High vs. Low)	1.58(0.97-2.58)	0.069	-	-
T stage (T2-T4 vs. Ta-T1)	1.54(0.77-3.10)	0.224	-	-
N stage (N1-3 vs. N0)	2.27(1.34-3.86)	**0.002**	1.84(1.07-3.17)	**0.028**
CD66b expression (High vs. Low)	2.48(1.54-3.97)	**<0.001**	2.22(1.37-3.61)	**0.001**

*Variables significantly associated with DFS by univariate analysis were adopted as covariates in multivariable analysis. Significant *P* values are shown in bold. HR > 1, risk for death increased; HR < 1, risk for death reduced.

Supplemental Table 6. Univariate and multivariate analysis of MPO expression and clinicopathological factors associated with disease-free survival (DFS) in bladder cancer from Cohort 1.

Prognostic parameters	Univariate analysis		Multivariate analysis*	
	HR (95% CI)	*P*	HR (95% CI)	*P*
Age (years) (≥65 vs. < 65)	1.19(0.74-1.90)	0.472	-	-
Gender (Male vs Female)	1.11(0.53-2.32)	0.777	-	-
Smoke (Yes vs. No)	1.19(0.75-1.89)	0.464	-	-
Tumor size (cm) (≥3 vs. < 3)	0.89(0.56-1.41)	0.617	-	-
Tumor number (≥2 vs. 1)	0.76(0.47-1.22)	0.249	-	-
Histological stage (High vs. Low)	1.58(0.97-2.58)	0.069	-	-
T stage (T2-T4 vs. Ta-T1)	1.54(0.77-3.10)	0.224	-	-
N stage (N1-3 vs. N0)	2.27(1.34-3.86)	**0.002**	1.91(1.08-3.36)	**0.026**
MPO expression (High vs. Low)	1.84(1.16-2.93)	**0.010**	1.54(0.93-2.54)	0.090

*Variables significantly associated with DFS by univariate analysis were adopted as covariates in multivariable analysis. Significant *P* values are shown in bold. HR > 1, risk for death increased; HR < 1, risk for death reduced.

Supplemental Table 7. Correlations between ETV4 expression levels and clinicopathological characteristics in 161 BCa cases from Cohort 3.

		ETV4 expression		
Characteristics	Cases	Low (%)	High (%)	*P*
Total	161	78(48.4)	83(51.6)	
Age(years)				0.784
<65	88	44 (56.4)	44 (53.0)	
≥65	73	34 (43.6)	39 (47.0)	
Gender				0.219
Female	30	11 (14.1)	19 (22.9)	
Male	131	67 (85.9)	64 (77.1)	
Smoke				0.129
No	77	32 (41.0)	45 (54.2)	
Yes	84	46 (59.0)	38 (45.8)	
Tumor size(cm)				0.953
<3	53	25 (32.1)	28 (33.7)	
≥3	108	53 (67.9)	55 (66.3)	
Tumor number				0.948
1	115	56 (73.7)	59 (72.0)	
≥2	43	20 (26.3)	23 (28.0)	
T stage				**0.014**
pTa-pT1	37	25 (32.1)	12 (14.5)	
pT2-pT4	124	53 (67.9)	71 (85.5)	
N stage				**<0.001**
pN0	110	66 (84.6)	44 (53.0)	
pN1-3	51	12 (15.4)	39 (47.0)	
Histological grade				**0.043**
Low	21	15 (19.2)	6 (7.2)	
High	140	63 (80.8)	77 (92.8)	
Progression				**0.009**
No	79	47 (60.3)	32 (38.6)	
Yes	82	31 (39.7)	51 (61.4)	
Alive status				**0.005**
Yes	88	52 (66.7)	36 (43.4)	
No	73	26 (33.3)	47 (56.6)	

Significant *P* values are shown in bold.

Supplemental Table 8. Univariate and multivariate analysis of ETV4 expression and clinicopathological factors associated with overall survival (OS) in bladder cancer from Cohort 3.

Prognostic parameters	Univariate analysis		Multivariate analysis*	
	HR (95% CI)	*P*	HR (95% CI)	*P*
Age (years) (≥65 vs. < 65)	1.50(0.95-2.37)	0.085	-	-
Gender (Male vs. Female)	0.91(0.49-1.69)	0.765	-	-
Smoke (Yes vs. No)	0.88(0.55-1.39)	0.571	-	-
Tumor size (cm) (≥3 vs. < 3)	1.46(0.87-2.44)	0.150	-	-
Tumor number (≥2 vs. 1)	1.07(0.64-1.80)	0.784	-	-
Histological stage (High vs. Low)	1.13(0.58-2.20)	0.723	-	-
T stage (T2-T4 vs. Ta-T1)	2.45(1.25-4.77)	**0.009**	1.51(0.74-3.08)	0.257
N stage (N1-3 vs. N0)	3.45(2.16-5.51)	**<0.001**	2.36(1.40-3.98)	**0.001**
ETV4 expression (High vs. Low)	2.88(1.76-4.69)	**<0.001**	2.06(1.21-3.48)	**0.007**

*Variables significantly associated with OS by univariate analysis were adopted as covariates in multivariable analysis. Significant *P* values are shown in bold. HR > 1, risk for death increased; HR < 1, risk for death reduced.

Supplemental Table 9. Univariate and multivariate analysis of ETV4 expression and clinicopathological factors associated with disease-free survival (DFS) in bladder cancer from Cohort 3.

Prognostic parameters	Univariate analysis		Multivariate analysis*	
	HR (95% CI)	*P*	HR (95% CI)	*P*
Age (years) (≥65 vs. < 65)	1.59(1.03-2.46)	**0.035**	1.56(1.01-2.42)	0.465
Gender (Male vs. Female)	1.01(0.56-1.84)	0.964	-	-
Smoke (Yes vs. No)	0.82(0.53-1.27)	0.385	-	-
Tumor size (cm) (≥3 vs. < 3)	1.34(0.83-2.15)	0.234	-	-
Tumor number (≥2 vs. 1)	1.01(0.62-1.66)	0.956	-	-
Histological stage (High vs. Low)	1.13(0.57-2.13)	0.713	-	-
T stage (T2-T4 vs. Ta-T1)	1.79(1.00-3.18)	**0.049**	1.09(0.58-2.04)	0.780
N stage (N1-3 vs. N0)	3.16(2.02-4.93)	**<0.001**	2.36(1.41-3.96)	**0.001**
ETV4 expression (High vs. Low)	2.38(1.51-3.76)	**<0.001**	1.77(1.07-2.93)	**0.027**

*Variables significantly associated with DFS by univariate analysis were adopted as covariates in multivariable analysis. Significant *P* values are shown in bold. HR > 1, risk for death increased; HR < 1, risk for death reduced.

Supplemental Table 10. List of reagents.

REAGENT	SOURCE	IDENTIFIER
Antibody		
CD66b	Zen BioScience	120405
MPO	Proteintech	66177-1-Ig
HOMO-LYVE1	Abcam	ab219556
MUS-LYVE1	Abcam	ab218535
Ly6G (1A8)	BioXcell	BE0075-1
Gr1	Cell Signaling Technology	31469
CXCR2	R&D Systems	MAB331
ETV4	Sigma-Aldrich	HPA005768
PTK6	Proteintech	18697-1-AP
p-ERK	Cell Signaling Technology	4370
p-JNK	Proteintech	80024-1-RR
E-cadherin	Proteintech	20874-1-AP
Snail	Cell Signaling Technology	3879
N-cadherin	Proteintech	22018-1-AP
VEGFA	R&D Systems	MAB293R
Flag	Proteintech	66008-3-Ig
Lamin B1	Proteintech	12987-1-AP
GAPDH	Proteintech	10494-1-AP
4G-10	Merck Millipore	05-321
anti-mus-CD45-FITC	Tonbo Biosciences	35-0451
anti-homo/mus-CD11b-APC	Tonbo Biosciences	20-0112
anti-mus-Ly6G-PE	Tonbo Biosciences	50-1276
anti-mus-F4/80-PE	eBioscience	12-4801-80
anti-homo-CD45-VF450	Tonbo Biosciences	75-0459
anti-homo-CD66b-FITC	eBioscience	11-0666-41
Ghost Dye™ Red 780	Tonbo Biosciences	13-0865
AF647 Donkey anti-goat	Bioss	bs-0294D-AF647
FITC Donkey anti-rabbit	Bioss	bs-0295D-FITC
Cy3 Donkey anti-mouse	Proteintech	SA00009
Inhibitor		
SB225002	MedChemExpress	HY-16711
Tilfrinib	MedChemExpress	HY-110244
MMP-9-IN-1	TargetMol	T8310
SP600125	Selleck	S2673
trameitinib	Selleck	S2673

Supplemental Table 11. List of primers used in this study.

Primer	Sequence
GAPDH-F	GTCTCCTCTGACTTCAACAGCG
GAPDH-R	ACCACCCTGTTGCTGTAGCCAA
ETV4-F	AGGAACAGACGGACTTCGCCTA
ETV4-R	CTGGGAATGGTCGCAGAGGTTT
CXCL1-F	AGCTTGCCTCAATCCTGCATCC
CXCL1-R	TCCTTCAGGAACAGCCACCAGT
CXCL8-F	GAGAGTGATTGAGAGTGGACCAC
CXCL8-R	CACAACCCTCTGCACCCAGTTT
VEGFA-F	TTGCCTTGCTGCTCTACCTCCA
VEGFA-R	GATGGCAGTAGCTGCGCTGATA
VEGFC-F	GCCAATCACACTTCCTGCCGAT
VEGFC-R	AGGTCTTGTTCGCTGCCTGACA
VEGFD-F	GACTGGAAGCTGTGGAGATGCA
VEGFD-R	GGCTGCACTGAGTTCTTTGCCA
MMP9-F	GCCACTACTGTGCCTTTGAGTC
MMP9-R	CCCTCAGAGAATCGCCAGTACT
CXCL2-F	GGCAGAAAGCTTGTCTCAACCC
CXCL2-R	CTCCTTCAGGAACAGCCACCAA
CXCL5-F	CAGACCACGCAAGGAGTTCATC
CXCL5-R	TTCCTTCCCGTTCTTCAGGGAG
CXCL6-F	GGGAAGCAAGTTTGTCTGGACC
CXCL6-R	AAACTGCTCCGCTGAAGACTGG
IL1B-F	CCACAGACCTTCCAGGAGAATG
IL1B-R	GTGCAGTTCAGTGATCGTACAGG
IL17A-F	CGGACTGTGATGGTCAACCTGA
IL17A-R	GCACTTTGCCTCCCAGATCACA
TNFA-F	CTCTTCTGCCTGCTGCACTTTG
TNFA-R	ATGGGCTACAGGCTTGTCACTC
CXCL1-ChIP-F	AGCTACCAGGTTAAATGA
CXCL1-ChIP-R	AGCACCCTTCCCACTGAA
CXCL8-ChIP-F	TGTCACTCCATGCACTGT
CXCL8-ChIP-R	GATGAATGAAGGTTTTCT

Supplemental Table 12. Sequences of siRNA oligos and shRNAs used in this study.

Name	Sequence 5'-3'
siRNA	
NC	UUCUCCGAACGUGUCACGUTT
si-ETV4#1	GCUGGAUGACCCAACAAAUTT
si-ETV4#2	CCCUCUUCUCUUUGGCCUUTT
si-PTK6	UGAAGAAGCUGCGGCACAATT
shRNA	
shCON.	CAACAAGATGAAGAGCACCAA
shETV4#1	GCTGGATGACCCAACAAAT

Supplemental Table 13. List of constructs information.

Construct	Sequence
CXCL1 promoter WT	AGTAATAAAGCTACCAGGTTAAATGACTGAAATTCCTGAGAGA AAACAACATGTGTGTGTTTCTCTAGAAAGGGGGCCCAATACTG AATACCAGGAAGTCCTATAGTAAATGGAATGTGACTCTATGTG GGATCCGGCGTTCCTATTTCATCCGAATGCATGTCTGCTGCTTC AGTGGGAAGGGTGCTTGCACACCAGGT
CXCL1 promoter MUT	AGTAATAAAGCTACCAGGTTAAATGACTGAAATTCCTGAGAG AAAACAACATGTGTGTGTTTCTCTAGAAAGGGGGCCCAATAC TGAATATTGAAGGACTCTATAGTAAATGGAATGTGACTCTAT GTGGGATCCGGCGTTCCTATTTCATCCGAATGCATGTCTGCTG CTTCAGTGGGAAGGGTGCTTGCACACCAGGT
CXCL8 promoter WT	CTCTTCTTGTCACTCCATGCACTGTGTTCCGTATGCTAAATAGT TTGAGAAACCCAAATGGGCCATGTTCGCCTACATTTCATTGTC CTGTACTTCCTGTCCTGTACTAGCAAAGCAGTCCCATTGGTCTT TCTTCTCCTCATTAACAATAAAGGTAACACTTTTGATGTTGTTT CTTCAGAAAACCTTCATTCATCAAA
CXCL8 promoter MUT	CTCTTCTTGTCACTCCATGCACTGTGTTCCGTATGCTAAATAGTT TGAGAAACCCAAATGGGCCATGTTCGCCTACATTTCATTGTCCT GCGTCCTTCACCCTGTACTAGCAAAGCAGTCCCATTGGTCTTTC TTCTCCTCATTAACAATAAAGGTAACACTTTTGATGTTGTTTCTT CAGAAAACCTTCATTCATCAAA
Primer of ETV4 Y392F (F)	GAACCGGCCAGCCATGAATTTCGACAAGCTGAGCCGCTCG
Primer of ETV4 Y392F (F)	CGAGCGGCTCAGCTTGTCGAAATTCATGGCTGGCCGGTTC
